# How Fit Are Special Operations Police Officers? A Comparison With Elite Athletes From Olympic Disciplines

**DOI:** 10.3389/fspor.2021.742655

**Published:** 2021-12-02

**Authors:** Lukas Zwingmann, Marvin Zedler, Stefan Kurzner, Patrick Wahl, Jan-Peter Goldmann

**Affiliations:** ^1^Department of Molecular and Cellular Sports Medicine, Institute of Cardiology and Sports Medicine, German Sport University Cologne, Cologne, Germany; ^2^The German Research Centre of Elite Sport Cologne, German Sport University Cologne, Cologne, Germany; ^3^Institute of Biomechanics and Orthopaedics, German Sport University Cologne, Cologne, Germany; ^4^Bureau for Education, Advanced Training, and Personnel Matters, North Rhine-Westphalia State Police, Selm, Germany

**Keywords:** fitness profile, policing, SWAT, maximum oxygen uptake (VO_2_max), occupational health, physical training and conditioning, elite athletes, performance testing

## Abstract

The diverse tasks of special operations police (SOP) units place high physical demands on every officer. Being fit for duty requires a wide range of motor abilities which must be trained regularly and in a structured manner. But SOP operators have to plan and manage large proportions of their training alone, which makes it difficult to control. Therefore, this study aimed to highlight strengths and deficits of the SOP operators' fitness by comparing them to elite athletes, and to define future training goals. Retrospective data of 189 male SOP operators were used, who completed several isometric strength tests, a graded exercise test to determine maximal oxygen uptake, and countermovement jumps to determine leg muscle power. On the basis of a literature search, performance data were then compared to a total of 3,028 elite male athletes from 36 Summer Olympic disciplines. Pooled means and standard deviations were calculated for each discipline and effect sizes were used to analyze their similarities and differences to the SOP unit. On average, SOP operators were taller, heavier, and stronger than elite athletes. But both the ability to convert this strength into explosive movement and aerobic power was significantly less developed. From this point of view, SOP operators should consider polarized endurance training to work efficiently on improving aerobic performance. In addition, regular plyometric training seems necessary to improve leg muscle power and agility.

## Introduction

Special operations police (SOP) units play a central role in the fight against high-risk crime and are primarily deployed in situations that exceed the competences and capabilities of general police authorities. The daily physical demands of SOP operators can vary greatly by nature and are related to the tasks being completed. A survey in Australia and New Zealand showed that the most common task is the execution of high-risk warrants of arrest, followed by rural operations, high-risk personal protection, and counter-terrorism response (Irving et al., 2019). SOP units are regularly challenged by physical activities such as running up or down stairs, pulling, lifting, and carrying heavy objects, running or sprinting over a variety of distances, and negotiating obstacles (Silk et al., 2018; Marins et al., 2020).

Apparently, police officers must have a high level of physical fitness, above average for general police service, when applying for a SOP unit. The importance of adequate and regular training is reinforced by the fact that individuals with good aerobic and muscular power show a higher success rate in selection courses (Hunt et al., 2013) and are less likely to get injured (Orr et al., 2013, 2016). Nevertheless, it is still somewhat difficult both to classify the physical requirements for SOP operators and to give applicants concrete recommendations for daily training. This is because few studies exist which have outlined a comprehensive fitness profile of SOP units (Pryor et al., 2012; Strader et al., 2020). Other research has examined only aerobic performance, but not muscular strength, agility, or body composition (Sperlich et al., 2011; Maupin et al., 2018). Contrasting, the functional capability and physical demands of general police units have widely been evaluated, showing a great heterogeneity between departments and countries (Marins et al., 2019).

Additionally, little is known about the training practices of SOP units, partly because they are often kept secret and partly because police officers have to design large proportions of their training programs autonomously (Davis et al., 2016; Marins et al., 2020). Marins et al. (2020) recently showed that the physical training of Brazilian tactical operators is to 48% unsupervised, 61% self-planned, and 50% unstructured, which is comparable to an earlier study by Davis et al. (2016) in the United States. Consequently, annual pass rates for SOP selection courses are highly variable, e.g., ranging from 18 to 70% in Australian special forces (Hunt et al., 2013). Therefore, accurately compiled performance standards and reference values to guide applicants and instructors might contribute to a more homogeneous selection process, less injuries, and thus to a more efficient use of human and financial resources.

Further, SOP units have to be fit and prepared for a variety of operations every day throughout the year. This requires not only the development of a wide range of motor abilities, but also a regular and structured training regimen. However, physical deficits are difficult to recognize because there are no internal references against which SOP operators can be measured. Using elite athletes as an external reference might allow to better classify and evaluate the metabolic and neuromuscular performance of SOP officers. Additionally, as much of the sporting activity is completed autonomously and takes place in leisure time as well, comparison to such individual or team athletes might be practical and easy to comprehend. Especially athletes from Olympic disciplines are widely studied and, therefore, training forms, volumes, and intensities can be derived from existing literature to optimize the SOP operators' daily physical preparation.

Accordingly, the purpose of this study is to provide a detailed fitness profile of a large cohort of German SOP operators with respect to anthropometry, aerobic performance, muscular strength, and vertical jump performance and to compare selected measures with those of elite athletes.

## Methods

### Participants

Anonymized retrospective data (2017–2020) were analyzed from a total of 189 German police officers who successfully completed the SOP selection process of the North Rhine-Westphalia State Police. All participants were male and in both cardiovascular and musculoskeletal health. The average body mass and height were 83.1 ± 7.1 kg and 182 ± 6 cm, respectively. To protect the identity of the police officers, no birth or age data were collected. However, the age of SOP candidates in Germany is usually between 25 and 35 years, as police officers have to resign from a SOP unit by the time they reach ~45 years (personal communication).

All participants were informed in detail about the examination procedures, techniques, and risks before signing the institutionally approved informed consent document. All procedures were approved by the Ethics Committee of the German Sport University Cologne under the number 051/2018 and were in accordance with the Declaration of Helsinki.

### Testing Procedures

All tests were performed on the same day and in randomized order, except for the initial bioelectrical impedance analysis and a 15-minute warm-up on a motorized treadmill at a self-selected speed. Participants were given a 30-min rest between the tests.

#### Anthropometrics

Body height was measured to the nearest 0.1 cm using a stadiometer (seca 274, seca GmbH & Co KG, Hamburg, Germany). Body mass, fat mass, and muscle mass were measured in standing position to an accuracy of 0.05 kg using a phase-sensitive 8-electrode bioelectrical impedance device (seca mBCA 515, seca GmbH & Co KG, Hamburg, Germany).

#### Muscle Strength

**Figure 1:**
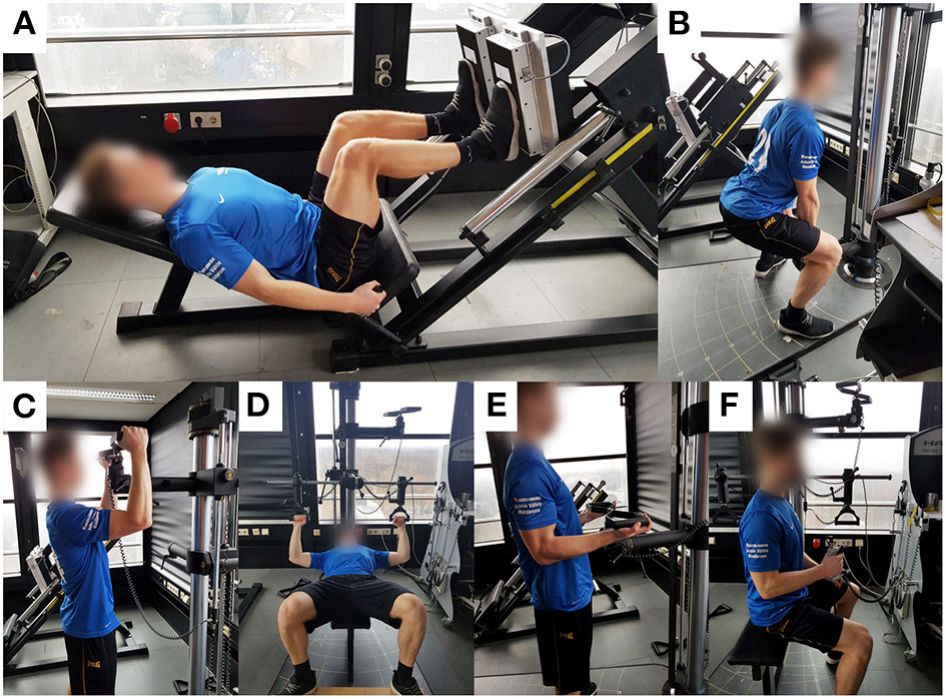
Pictures of the isometric strength test settings. **(A)** Leg press; **(B)** Upright pull; **(C)** High lift; **(D)** Bench press; **(E)** Arm lift; **(F)** Hand grip strength.

A total of six isometric strength tests were performed to give a holistic picture of whole-body muscle strength. To ensure high practical relevance and generalizability, the tests have been adopted from real physical tasks frequently encountered on duty such as (overhead) lifting, carrying, and grabbing (Pryor et al., 2012; Marins et al., 2020). Participants were verbally motivated to push or pull on a stationary force transducer (EvalTech, BTE Technologies, Hanover, NH, USA) with maximal effort. During each trial, the participants were instructed to slowly build up force and hold for 5 s once they reached maximal force. A trial was considered valid upon visual inspection of the force-time plateau. Each participant completed three trials per test of which the best was included in the analysis. A minimum of 2 min rest was allowed between each trial to reduce the risk of muscular fatigue. Isometric strength was determined in N and calculated using a 5 s average of the force-time plateau. Figure 1 shows the corresponding test settings.

##### Leg Press

The participants were placed in a leg press machine which was adjusted and fixed at a 90° knee angle. A custom-made force platform with strain gauges (Feinmechanische Entwicklungswerkstatt, German Sport University Cologne, Germany) was mounted onto the foot panel. The feet were positioned at hip width and in the middle of the panel.

##### Upright Pull

Participants were encouraged to pull on a force transducer (EvalTech, BTE Technologies, Hanover, USA) mounted at a height of 38 cm. This force transducer was also used for the high lift, bench press, and arm lift. The following criteria regarding lifting technique had to be fulfilled: (1) Fixation of the spinal column in a neutral position, (2) positioning of the shoulders vertically above the line of action, (3) avoidance of flexion in the elbow joint or lifting the heel off the ground.

##### High Lift

Participants had to push vertically upwards on the force transducer mounted above shoulder height. To do this, they stood upright, legs extended, upper arms raised at 90° anteversion, and elbow joints flexed at 90°. The heels were not allowed to be lifted from the ground for a valid attempt.

##### Bench Press

The participants lay with their backs on a bench and had to press on two separate handles mounted at a height and width at which the upper arms were abducted 90° and the forearms positioned in vertical direction resulting in 90° flexion of the elbow joints. The buttocks had to be in contact with the bench during each attempt.

##### Arm Lift

This test was performed in a standing position. The handles were set at a height at which the elbow joints were bent 90° without shoulder anteversion. With the forearms in supination, the subjects again had to pull vertically upwards on the handles. For a valid test, the heels and shoulders were not allowed to be lifted.

##### Hand Grip Strength

Hand grip strength was measured in a sitting position for the dominant and non-dominant hand (Trailite, LiteXpress GmbH, Ahaus, Germany). The elbow joints were in 90° flexion and the forearms in neutral position. For further analyses, the best trials of both hands were averaged.

#### Vertical Jump Performance

Each participant performed countermovement jumps (CMJ) without arm swing and hands placed on the hips. Jump height was defined as the highest out of five trials and calculated by mathematical integration of the force-time curve of the vertical ground reaction force (MATLAB, The MathWorks Inc., Natick, MA, USA), which was recorded by means of two force plates (Model 9281B, 1,000 Hz, Kistler Instrumente AG, Winterthur, Switzerland).

#### Aerobic Performance

An incremental exercise test on a motorized treadmill (saturn®, h/p/cosmos sports & medical GmbH, Nussdorf-Traunstein, Germany) was performed, which started at a speed of 2.4 m·s^−1^ and increased by 0.4 m·s^−1^ every 5 min until volitional exhaustion was reached. A 30 s rest was given between increments to allow capillary blood sampling from the earlobe. VO_2_ was measured using a stationary breath by breath gas analyser (MetaLyzer 3B, Cortex Biophysik GmbH, Leipzig, Germany), and VO_2_max was defined as the highest 30 s moving average. Blood lactate concentrations were measured using an enzymatic amperometric analyser (Biosen S-Line, EKF Diagnostics, Cardiff, Wales), plotted against running velocity, and fitted by a third order polynomial function. Afterward, anaerobic threshold (second lactate threshold, LT2) was calculated using the modified maximal deviation method (Zwingmann et al., 2019).

### Literature Search

#### Search Strategies

To classify the fitness profile of SOP operators in the context of elite sports, a literature search was conducted in PubMed and Google Scholar databases during a period from November 2020 to February 2021. As the methodology of studies conducting performance tests often considerably differs (especially with regard to strength tests), the literature search focused on the parameters body mass, body height, VO_2_max, hand grip strength, and CMJ height. The search terms consisted of all current Summer Olympic disciplines, each of which was combined with one or more of the following terms using the Boolean operators AND and OR: fitness, physiological profile, aerobic capacity, aerobic power, VO_2_max, grip strength, vertical jump, countermovement jump, CMJ. In addition, the reference lists of reviews and selected studies as well as the lists of similar articles provided by the databases were searched for further eligible literature.

#### Selection Criteria and Data Extraction

To ensure that the physical performance reported in the selected studies is consistent with the current level of elite athletes, only peer-reviewed publications from 2010 or later were included. They had to be written in English and available as full text. Additionally, information had to be provided in the manuscript that the athletes were adult males and performed their sport at professional level.

Information on at least one of the chosen performance parameters (mean, SD, sample size) and the methods used had to be provided. If the results were only presented graphically, mean ± SD were extracted using a web-based plot digitizer (WebPlotDigitizer; https://automeris.io/WebPlotDigitizer/).

VO_2_max had to be measured by means of an exhaustive graded exercise test using a gas analyser. The CMJ height had to be determined either by integrating the force-time curve of the vertical ground reaction force or by flight time. Hand grip strength had to be recorded isometrically and at least with the dominant hand.

If a parameter was determined in multiple studies, the means and SD for each sport were combined using the formulas proposed by Higgins et al. (2020):


(1) $$\begin{array}{l} Mean=\;\frac{N_{1}M_{1}+\;N_{2}M_{2}}{N_{1}+\;N_{2}} \end{array}$$

(2) $$\begin{array}{l} SD=\;\sqrt{\frac{\left(N_{1}-1\right)SD_{1}^{2}+\;\left(N_{2}-1\right)SD_{2}^{2}+\frac{N_{1}N_{2}}{N_{1}+\;N_{2}}\left(M_{1}^{2}+\;M_{2}^{2}-2M_{1}M_{2}\right)\;}{N_{1}+\;N_{2}-1}} \end{array}$$

where N_1_, N_2_ are the sample sizes, SD_1_, SD_2_ are the standard deviations, and M_1_, M_2_ are the means of the two studies to be combined. For more than two studies, the formulas were applied stepwise. Detailed information about the studies included are provided as Supplementary Material.

### Statistical Analysis

Descriptive statistics of the data are presented as means ± SD. Coefficients of variation (CV) were calculated to better describe the variability of the parameters recorded. To compare the outcomes between sports and the SOP unit, adjusted effect sizes (*d*) were calculated as proposed by Hedges and Olkin (1985). *d* smaller 0.35 was considered a trivial effect, *d* between 0.35 and 0.8 a small effect, *d* between 0.8 and 1.5 a moderate effect, and *d* greater 1.5 a strong effect (Bernards et al., 2017).

## Results

The anthropometric and performance data of the SOP unit are presented in Table 1.

**Table 1:** Anthropometric and performance test results of German special operations police operators.

**Parameter**	**N**	**Mean ± SD**	**CV (%)**	**95% Confidence Interval**	**Range**
**Anthropometrics**					
Body mass (kg)	177	83.1 ± 7.1	8.5	82.1–84.1	66.1–111.1
Body height (cm)	177	182.4 ± 6.0	3.3	181.5–183.3	169.5–202.5
Fat mass (kg)	177	11.0 ± 3.6	32.7	10.5–11.5	2.3–24.4
Fat mass (%)	177	13.1 ± 3.6	27.5	12.6–13.6	3.0–22.6
Lean mass (kg)	177	72.1 ± 5.3	7.4	71.3–72.9	61.2–86.7
Lean mass (%)	177	86.8 ± 3.6	4.1	86.3–87.4	79.5–97.0
Total muscle mass (kg)	177	35.9 ± 2.9	8.1	35.5–36.3	30.1–43.6
Muscle mass torso (kg)	177	16.8 ± 1.4	8.3	16.6–17.0	13.2–20.5
Muscle mass lower limbs (kg)	177	14.0 ± 1.2	8.6	13.8–14.2	11.2–16.9
Muscle mass upper limbs (kg)	177	5.1 ± 0.5	9.8	5.0–5.2	4.0–6.6
**Muscle strength**					
Leg press (N)	177	*2, 580*±456	17.7	2,573–2,587	1,686–4,044
Upright pull (N)	177	*1, 401*±199	14.2	1,372–1,431	830–2,032
High lift (N)	177	790 ± 214	27.1	759–822	440–1,662
Bench press (N)	177	*1, 128*±164	14.6	1,104–1,152	789–1,579
Arm lift (N)	177	549 ± 75	13.7	538–560	336–873
Hand grip strength (N)	177	549 ± 94	17.0	535–563	308–831
**Vertical jump performance**					
CMJ (cm)	177	36.8 ± 5.1	13.9	36.0–37.6	22.6–51.2
**Aerobic power**					
VO_2_max (L·min^−1^)	189	4.35 ± 0.44	10.2	4.29–4.41	3.13–5.70
VO_2_max (mL·min^−1^·kg^−1^)	189	52.4 ± 4.1	7.8	51.8–53.0	41.0–66.5
LT2 (m·s^−1^)	183	3.57 ± 0.22	5.6	3.54–3.60	2.84–4.28

*CV, Coefficient of Variation; CMJ, Countermovement jump; LT2, Second lactate threshold; VO_2_max, Maximum oxygen uptake*.

As a result of the literature search, a total of 138 studies met the criteria for inclusion in the analyses. These studies reported performance data from 3,028 professional male athletes participating in one of 36 Summer Olympic sports or disciplines. No studies could be included for the following Summer Olympic sports or disciplines due to either inappropriate methodology or sampling: Archery, Decathlon, Discus Throw, Javelin Throw, Shot Put, Long Jump, Pole Vault, Triple Jump, Breaking, Track Cycling, Diving, Equestrian, Skateboarding, and Trampoline.

**Figure 2:**
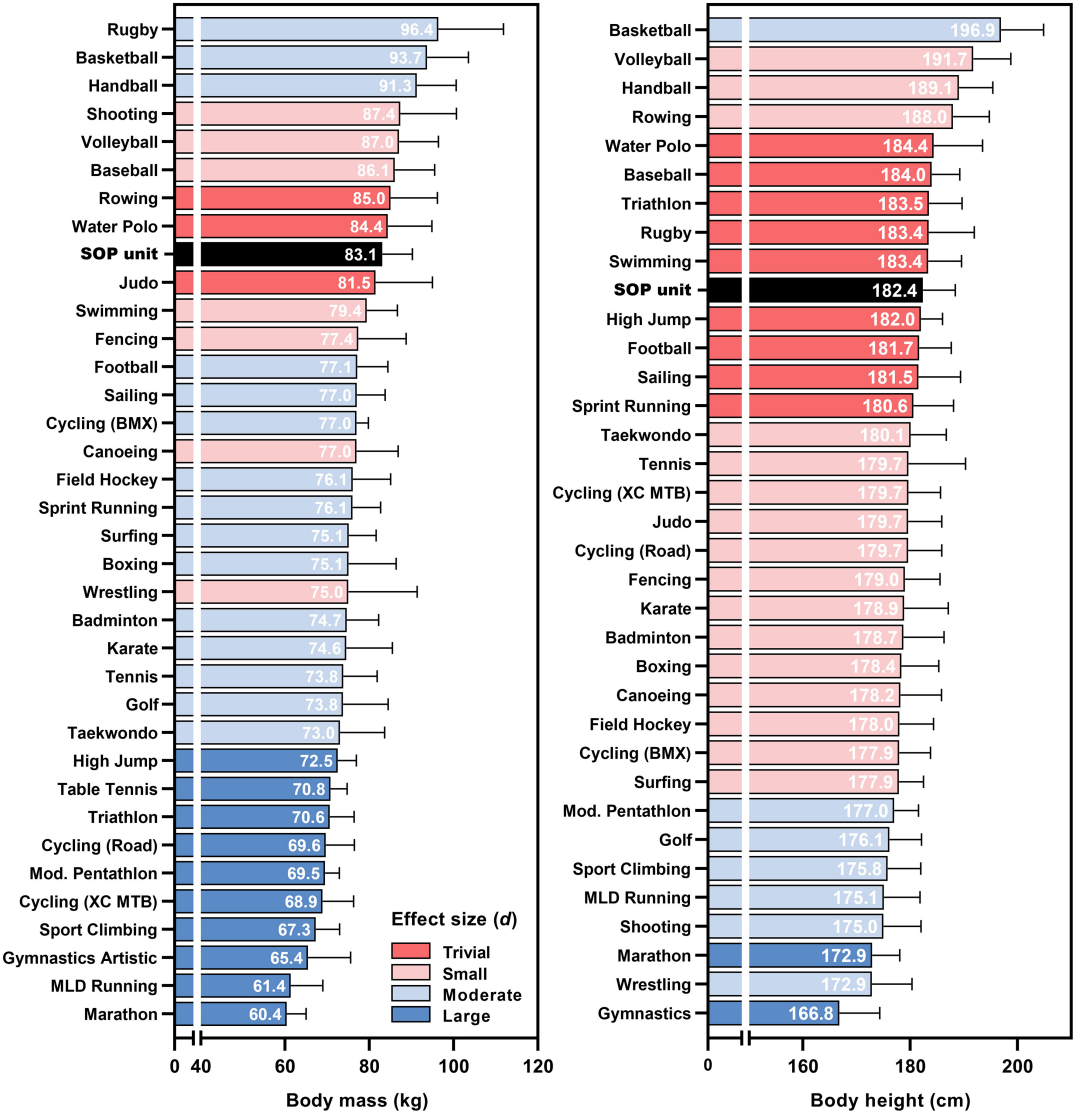
Average body mass and height of elite athletes from Summer Olympic disciplines and the Special Operations Police unit (SOP unit), sorted by size. The color scale illustrates the effect size of each discipline compared to the SOP unit. BMX, Bicycle motocross; MLD, Middle- and long distance; XC MTB, Cross-country mountain bike.

For body mass and body height, 2,889 and 2,694 athletes from 35 and 34 sports were included. Rankings of the combined means ± SD for each cohort and parameter (including the SOP unit) are shown in Figure 2. The resulting differences between each sport and the SOP unit, as well as the effect sizes are provided in Table 2. The SOP unit was among the 10 heaviest and tallest cohorts analyzed. Following the calculated effect sizes, body mass of the SOP unit was most similar to judoka, water polo players and rowers, with *d* between −0.18 and 0.22. Marathon runners were the lightest cohort (60.4 ± 4.6 kg, *d* = −3.32), rugby players were the heaviest cohort analyzed (96.4 ± 15.4 kg, *d* = 1.16). Trivial effect sizes regarding body height were found in seven cohorts ranging from−0.29 to 0.28, including sports like football, high jumping, swimming, and rugby. Gymnasts were the smallest (166.8 ± 7.5 cm, *d* = −2.46), basketball players were the tallest cohort analyzed (196.9 ± 7.9 cm, *d* = 2,09).

**Table 2:** Number of athletes included per sport, Coefficient of Variation (CV) within sport, difference between the mean of each sport and the special operations police unit, and effect size (*d*) for body mass and body height.

**Sport/discipline**	**Body mass (kg)**				**Body height (cm)**			
	**N**	**CV (%)**	**Diff**.	***d***	**N**	**CV (%)**	**Diff**.	***d***
Athletics: high jump	5	6.07	−10.60	−1.50	5	2.20	−0.40	−0.07
Athletics: marathon	29	7.60	−22.72	−3.32	29	2.96	−9.47	−1.60
Athletics: MLD running	119	12.38	−21.72	−2.97	80	3.83	−7.31	−1.17
Athletics: sprint running	35	8.74	−7.05	−1.00	35	4.12	−1.79	−0.29
Badminton	82	10.07	−8.43	−1.16	82	4.24	−3.68	−0.56
Baseball	190	10.95	2.96	0.35	190	2.86	1.59	0.28
Basketball	146	10.50	10.59	1.25	146	4.02	14.54	2.09
Boxing	40	15.00	−8.01	−1.00	40	3.91	−4.05	−0.65
Canoeing	46	12.80	−6.14	−0.79	46	4.29	−4.20	−0.66
Cycling (BMX)	16	3.64	−6.14	−0.89	16	3.29	−4.47	−0.74
Cycling (Road)	97	9.72	−13.46	−1.92	81	3.47	−2.75	−0.45
Cycling (XC MTB)	18	10.68	−14.18	−1.98	18	3.33	−2.73	−0.45
Fencing	59	14.72	−5.74	−0.68	49	3.64	−3.38	−0.55
Field hockey	47	11.82	−7.03	−0.93	47	3.56	−4.40	−0.72
Football	488	9.45	−5.97	−0.82	470	3.27	−0.72	−0.12
Golf	31	14.43	−9.32	−1.20	31	3.40	−6.29	−1.05
Gymnastics (Artistic)	49	15.42	−17.67	−2.25	40	4.50	−15.58	−2.46
Handball	139	10.20	8.20	1.00	139	3.32	6.69	1.09
Judo	53	16.52	−1.62	−0.18	53	3.47	−2.75	−0.45
Karate	66	14.65	−8.55	−1.03	57	4.57	−3.50	−0.53
Modern pentathlon	7	4.88	−13.60	−1.93	7	2.58	−5.40	−0.90
Rowing	101	13.06	1.94	0.22	101	3.60	5.58	0.89
Rugby	133	15.98	13.31	1.16	69	4.63	1.03	0.15
Sailing	36	8.67	−6.06	−0.86	36	4.31	−0.88	−0.14
Shooting	46	15.22	4.28	0.49	46	4.00	−7.40	−1.19
Sport climbing	36	8.40	−15.81	−2.29	36	3.52	−6.61	−1.09
Surfing	31	8.68	−7.99	−1.13	31	2.55	−4.48	−0.77
Swimming	146	9.13	−3.70	−0.52	146	3.37	0.97	0.16
Table tennis	11	5.51	−12.30	−1.76				
Taekwondo	67	14.51	−10.06	−1.22	56	3.69	−2.33	−0.38
Tennis	82	10.82	−9.27	−1.25	82	5.91	−2.72	−0.35
Triathlon	26	8.16	−12.49	−1.79	18	3.36	1.10	0.18
Volleyball	141	10.77	3.94	0.48	141	3.65	9.33	1.44
Water polo	172	12.40	1.27	0.14	172	4.93	1.96	0.25
Wrestling	99	21.75	−8.08	−0.71	99	4.31	−9.53	−1.45

*BMX, Bicycle motocross; MLD, Middle- and long-distance; XC MTB, Cross-country mountain bike*.

**Figure 3:**
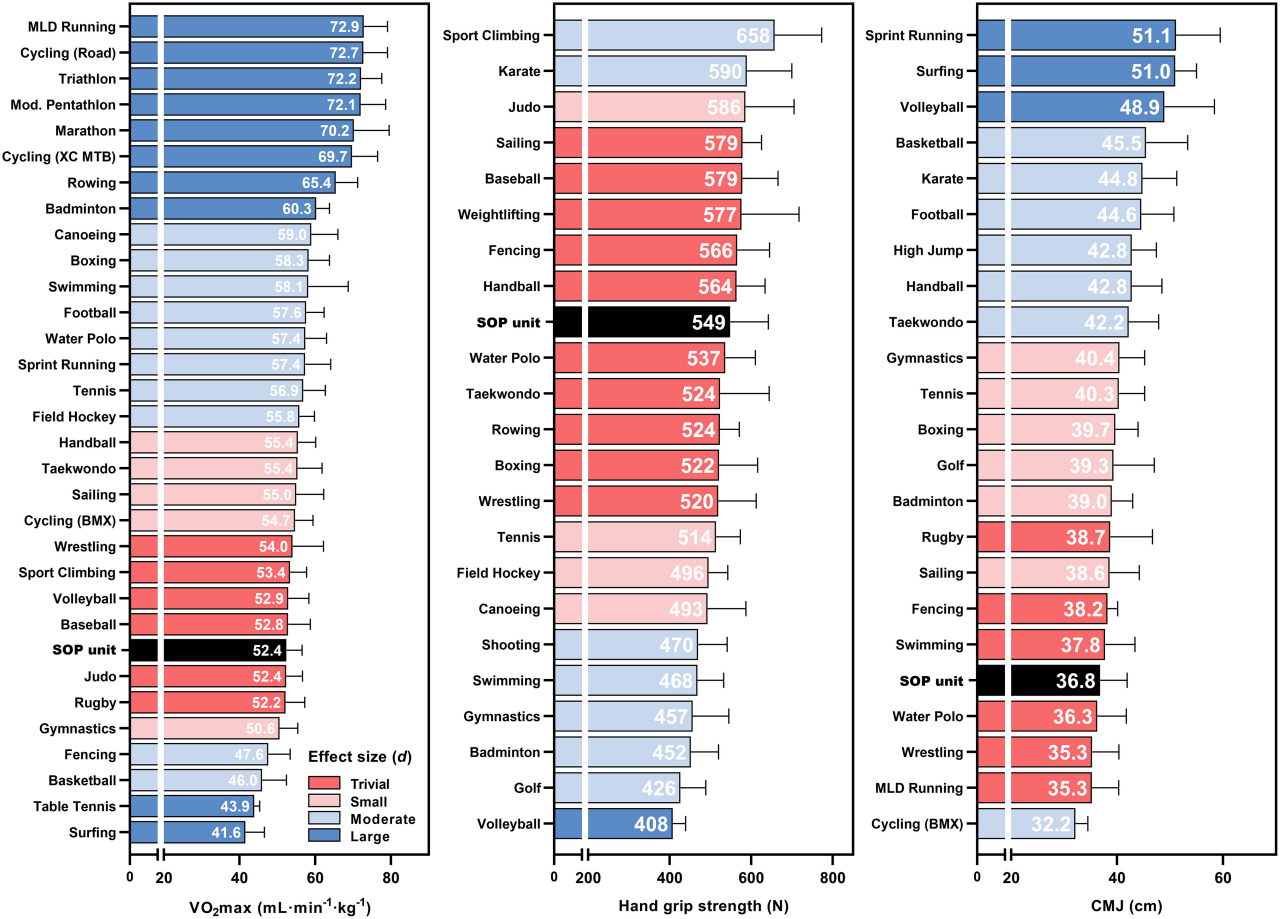
Average maximum oxygen uptake (VO_2_max), hand grip strength, and countermovement jump height (CMJ) of elite athletes from Summer Olympic disciplines and the Special Operations Police unit (SOP unit), sorted by size. The color scale illustrates the effect size of each discipline compared to the SOP unit. BMX, Bicycle motocross; MLD, Middle- and long distance; XC MTB, Cross-country mountain bike.

The number of athletes included in the analyses of VO_2_max, hand grip strength, and CMJ was 1,599, 815, and 1,421, each of whom participated in one of 31, 23, and 23 sports or disciplines, respectively. Rankings of the combined means ± SD for each cohort and parameter (including the SOP unit) are shown in Figure 3. The resulting differences between each sport and the SOP unit, as well as the effect sizes are provided in Table 3. With 52.4 ± 4.1 mL·min^−1^·kg^−1^, VO_2_max of the SOP unit was among the eight weakest cohorts analyzed. The highest values were found in middle- to long-distance runners (72.9 ± 6.2 mL·min^−1^·kg^−1^, *d* = 4.20), the lowest values were found in surfers (41.6 ± 5.0 mL·min^−1^·kg^−1^, *d* = −2.57). The VO_2_max of SOP operators was most similar to that of rugby, baseball, volleyball players, judoka, and sport climbers (*d* ranging from −0.06 to 0.33).

**Table 3:** Number of athletes included per sport, Coefficient of Variation (CV) within sport, difference between the mean of each sport and the special operations police unit, and effect size (*d*) for maximum oxygen uptake (VO_2_max), hand grip strength and countermovement jump height (CMJ).

**Sport/discipline**	**VO**_**2**_ **max (mL·min** ^**−1**^ **·** **kg** ^**−1**^ **)**				**Hand grip strength (N)**				**CMJ (cm)**			
	**N**	**CV (%)**	**Diff**.	***d***	**N**	**CV (%)**	**Diff**.	***d***	**N**	**CV (%)**	**Diff**.	***d***
Athletics: high jump									5	10.75	6.00	1.17
Athletics: marathon	29	13.25	17.84	3.50								
Athletics: MLD running	85	8.56	20.46	4.20					34	14.33	−1.55	−0.30
Athletics: sprint running	24	11.72	4.95	1.11					41	16.29	14.33	2.45
Badminton	45	5.81	7.87	1.97	62	14.97	−96.80	−1.10	62	10.02	2.22	0.46
Baseball	124	11.19	0.40	0.08	66	15.08	29.59	0.32				
Basketball	69	13.96	−6.41	−1.32					146	17.22	8.67	1.33
Boxing	30	9.44	5.86	1.35	10	18.10	−27.29	−0.29	20	10.78	2.89	0.57
Canoeing	65	11.87	6.58	1.31	23	19.00	−55.60	−0.59				
Cycling (BMX)	6	8.59	2.30	0.56					10	7.25	−4.65	−0.93
Cycling (Road)	97	8.73	20.32	4.07								
Cycling (XC MTB)	18	9.64	17.34	3.95								
Fencing	49	12.15	−4.81	−1.07	24	14.04	16.84	0.18	10	4.97	1.40	0.28
Field hockey	17	7.17	3.40	0.83	30	9.52	−53.37	−0.60				
Football	407	8.25	5.19	1.14					448	13.85	7.76	1.32
Golf					18	14.65	−122.71	−1.33	23	19.56	2.52	0.46
Gymnastics (Artistic)	11	9.49	−1.80	−0.43	19	19.31	−92.01	−0.98	38	11.72	3.64	0.72
Handball	38	8.50	3.02	0.72	32	12.35	15.22	0.17	107	13.23	5.99	1.12
Judo	10	8.16	−0.05	−0.01	53	20.36	37.05	0.37				
Karate					26	18.65	40.65	0.42	48	14.39	8.02	1.48
Modern pentathlon	7	9.13	19.65	4.66								
Rowing	86	8.88	13.02	2.76	27	9.05	−25.44	−0.28				
Rugby	18	9.70	−0.25	−0.06					108	20.56	1.91	0.30
Sailing	36	13.19	2.58	0.54	21	8.04	30.08	0.33	21	14.51	1.80	0.35
Shooting					46	15.15	−79.07	−0.88				
Sport climbing	20	8.19	0.96	0.23	23	17.56	108.65	1.12				
Surfing	21	12.03	−10.84	−2.57					10	7.84	14.20	2.80
Swimming	66	18.18	5.73	0.89	59	13.77	−80.71	−0.92	30	14.89	0.95	0.18
Table tennis	11	3.19	−8.50	−2.11								
Taekwondo	43	11.69	2.95	0.64	35	22.98	−25.06	−0.25	23	13.29	5.41	1.04
Tennis	41	10.18	4.47	1.00	8	11.64	−35.13	−0.38	44	12.05	3.52	0.69
Triathlon	26	7.54	19.75	4.60								
Volleyball	64	10.33	0.48	0.11	30	7.62	−141.04	−1.60	90	19.32	12.12	1.76
Water polo	8	9.76	5.00	1.20	107	13.74	−12.20	−0.14	57	15.12	−0.55	−0.11
Weightlifting					25	24.46	27.63	0.27				
Wrestling	28	15.19	1.60	0.33	71	17.80	−29.32	−0.31	46	14.32	−1.53	−0.30

*BMX, Bicycle motocross; MLD, Middle- and long-distance; XC MTB, Cross-country mountain bike*.

Hand grip strength showed less variability between cohorts than the other parameters analyzed. The SOP unit was above average (549 ± 94 N) and showed low effect sizes against 10 sports (*d* ranging from −0.31 to 0.33). Sport climbers showed the highest grip strength (658 ± 116 N, *d* = 1.12), volleyball players the lowest (408 ± 31 N, *d* = −1.60).

CMJ height of the SOP unit was among the five lowest cohorts analyzed (36.8 ± 5.1 cm). The biggest difference to the SOP unit was shown by the sprint runners who jumped about 14.33 cm higher on average (*d* = 2.45). Effect sizes were trivial in comparison to middle- and long-distance runners, wrestlers, water polo players, swimmers, and fencers (*d* ranging from −3.00 to 3.00).

## Discussion

The primary purpose of this study was to describe the physical characteristics and performance of German SOP operators and to compare selected parameters with those of professional athletes.

In terms of body anthropometry, SOP operators were shown to be heavier and taller than the average of all cohorts. With 182.4 ± 6.0 cm, body height was most similar to that of football players, high jumpers, swimmers, or rugby players, but smaller than that of basketball, volleyball, handball players, or rowers. In some sports, such as those previously mentioned, a tall body height offers several advantages in terms of athletic performance (Tsoukos et al., 2019; Zarić et al., 2020). The importance of body height in the police profession has been subject to a controversial debate for decades. Internationally, a wide range of minimum height requirements exist as a selection criterion for police departments, varying from 152 cm in Belgium to 170 cm in Greece (Kirchengast, 2010). In the German state of North Rhine-Westphalia, this limit is currently 163 cm for both men and women (Ministry of the Interior of North Rhine-Westphalia, 2021). In some countries, such minimum requirements have been abolished because of their discriminatory nature and the lack of scientific evidence (Anderson et al., 2001; Kirchengast, 2010). To date, a few studies have found a weak relationship between body height and occupational task performance (Orr et al., 2018; Zwingmann et al., 2021b). In the study of Orr et al. (2018), taller participants tended to be more successful on the Australian Specialist Selection Course. Participants employed for the specialist unit averaged 183.8 ± 4.6 cm, which is similar to the average body height found in the present study. Consequently, body height appears to be a non-negligible factor in the police profession, similar to many Olympic sports reported in the present study. According to earlier research, however, the body height of SOP operators does not differ significantly from male officers of general police authorities in western countries (Leischik et al., 2015; Dawes et al., 2017).

Since increasing body height is accompanied by increasing body mass, it is not surprising that 8 of the 10 tallest cohorts analyzed here are also among the 10 heaviest cohorts, including the SOP unit. With trivial effect sizes, body mass of SOP operators was most similar to that of rowers, water polo players, and judokas. Since elite athletes “have body sizes and compositions that closely match the specific constraints of their disciplines” (Haugen et al., 2018), this similarity could mainly come from the fact that in all these sports a large absolute strength and power output is crucial during physical contact with opponents or to accelerate heavy objects, such as a skull. Body mass, especially lean body mass, and a mesomorphic somatotype have not only been shown to positively correlate with power output in rowers and judokas (Nevill et al., 2010; Lewandowska et al., 2011), but also with intensive policing activities, such as heavy lifting, dragging, or carrying (Vanderburgh, 2008; Zwingmann et al., 2021b). Contrary, increased body mass is associated with impaired endurance performance and increased energy expenditure (Vanderburgh, 2008; Zwingmann et al., 2021a,b). Therefore, and with respect to the varying physical demands of SOP units, it might not be a maximum but an optimum body size that is decisive for operational capability, roughly ranging from 181 to 184 cm and 81 to 85 kg when compared to elite athletes.

In this context, the contribution of fat and muscle mass on total body mass needs to be considered as well. Using dual-energy X-ray absorptiometry, these anthropometric traits have been shown to average 10.9 kg (13.9%) and 64.6 kg (84.9%), respectively, in a male athletic population (Santos et al., 2014). The SOP operators' average fat and lean mass were 11.0 kg (13.1%) and 72.1 kg (86.8%). Despite a high variability of fat mass (CV = 32.7%), this indicates their athletic physique and an above-average muscle mass. Santos et al. (2014) found the greatest lean mass in handball (69.2 kg, 82.7%) and basketball players (68.5 kg, 83.6%). In rowers, wrestlers/judokas, and other combat sports athletes, lean mass was 65.4 kg (83.3%), 61.2 kg (85.5%), and 59.9 kg (85.2%), respectively. But it must be noted that given differences to the present study might partially be due to a wider range of the participants' age (16–45 years), smaller body height, and different measurement methods.

Measures of muscle strength are somewhat difficult to compare with other studies, because the type of muscle work (concentric dynamic, isokinetic, isometric) as well as the joint angles chosen greatly influence their outcomes. Nevertheless, for instance, the bench press one repetition maximum has been shown to average 88 ± 14 to 102 ± 13 kg in elite basketball players (Ben Abdelkrim et al., 2010; Balsalobre-Fernández et al., 2014), 89 ± 19 kg in karate athletes (Loturco et al., 2014), 91 ± 22 to 105 ± 18 kg in canoeists (Hamano et al., 2015), and 145 ± 5 to 164 ± 5 kg in judokas (Drid et al., 2015). Loturco et al. (2016) demonstrated a bench and leg press isometric strength of ~1,018 ± 26 and 2,610 ± 951 N, respectively, in elite boxers using comparable joint angles as in the present study. Hence, with 1,128 ± 164 and 2,580 ± 456 N, bench and leg press isometric strength of SOP operators was equivalent to that of combat sports athletes, although no eligible studies exist to provide information on other sports.

One strength parameter that has been evaluated more frequently under comparable test conditions is hand grip strength. In many movement patterns, such as throwing or hitting, the hands represent the end of a kinetic chain through which a sum of muscular forces and resultant joint moments is transferred to an external sporting device or opponent. The significance of hand grip strength as a limiting determinant of movement performance has repeatedly been demonstrated in sports such as climbing, baseball, or wrestling (Cronin et al., 2017). In the case of SOP units, fighting techniques, climbing tasks, and fast-roping from helicopters are activities that require a certain degree of hand grip strength as well. Further, hand grip strength is a weak but significant correlate of marksmanship with firearms (Copay and Charles, 2001). Although the influence of specific grip strength training on athletic performance is not well-documented, it appears that grip strength is a covariate of overall muscle strength in a variety of sports and, therefore, positively affected by regular whole-body resistance training (Cronin et al., 2017).

In this study, hand grip strength values ranged from 408 ± 31 N (volleyball players) to 658 ± 115 N (sport climbers). With 549 ± 94 N, the SOP unit was among the 10 strongest cohorts and most similar to water polo players, handball players, and fencers, with a difference of <20 N. But effect sizes were also trivial compared to wrestlers, boxers, rowers, taekwondo athletes, weightlifters, baseball players, and sailors. These similarities seem plausible primarily because all of these Olympic disciplines involve an external sporting device or physical contact with opponents. As a limitation, however, it needs to be mentioned that in some cohorts either very small samples were examined or a high coefficient of variation led to small effect sizes (Table 3).

Overall, the shown strength test results indicate that a large proportion of the SOP operators' athletic training consists of heavy resistance exercises, which is in line with previous publications (Pryor et al., 2012; Davis et al., 2016; Marins et al., 2020). Contrasting, CMJ height was among the five worst performing cohorts, indicating a certain training deficit regarding leg muscle power. CMJ height of SOP operators was most similar to swimmers and Water Polo players. While the upper extremities are predominantly performance-limiting in such aquatic sports, the need for high dynamic leg power and agility in many policing activities seems intuitive and has frequently been demonstrated (Mala et al., 2015; Moreno et al., 2019). Regular plyometric and sprint training is known to improve such motor abilities (Markovic et al., 2007), which is emphasized in the present study by the fact that sprint runners showed the best CMJ performance, followed by sports like volleyball and basketball. Therefore, a higher amount of plyometric and sprint training, as performed by sprinters or volleyball players (Haugen et al., 2019; Silva et al., 2019), should be considered for SOP training programs.

Aerobic power is another physical characteristic that is classified as crucial in police forces. Studies show that a high VO_2_max positively affects operational task performance (Sperlich et al., 2011; Hunt et al., 2013; Zwingmann et al., 2021b) as well as injury prevalence (Orr et al., 2013). In competitive sports, it is believed to allow for improved recovery (Tomlin and Wenger, 2001) and to be an important requirement for gains in training volume and intensity. In men, VO_2_max can reach very different magnitudes. Average values in healthy European males are highly variable and range from 25 to 51 mL·min^−1^·kg^−1^, depending on age and physical activity (Van Der Steeg and Takken, 2021; Wagner et al., 2021). Contrarily, absolute and relative values in elite athletes can reach 7 L·min^−1^ and 85 mL·min^−1^·kg^−1^ or higher (Haugen et al., 2018). In this study, relative VO_2_max in most cohorts was between 50 and 60 mL·min^−1^·kg^−1^, including the SOP unit (52.4 ± 4.1 mL·min^−1^·kg^−1^). Especially team, racket, and combat sports fall into this range, which are often characterized as sports with intermittent metabolic demands and frequent changes of direction (Chaabène et al., 2015; Fernandez-Fernandez et al., 2015; Peña et al., 2018). Overall, however, SOP operators were among the 10 worst performing cohorts analyzed here. With a trivial effect size, they were most similar to rugby, baseball, volleyball players, wrestlers, sport climbers, and judokas. It is notable that four of these six sports as well as the SOP unit were also among the 10 heaviest cohorts, illustrating the general body mass penalty of aerobic fitness tests against heavier individuals (Vanderburgh, 2008). However, consistent with previous research (Pryor et al., 2012), it shows that the potential in the development of VO_2_max is not yet fully utilized by SOP units. Although police officers frequently report a high proportion of regular endurance training (Davis et al., 2016; Irving et al., 2019; Marins et al., 2020), the training regimen might not yet be as effective or efficient as in most Olympic sports. On the other hand, it is questionable whether SOP operators can even achieve a comparable training volume and aerobic power like elite athletes who practice full-time and are able focus significantly more on performance optimization. Nevertheless, other studies on specialist forces revealed high VO_2_max values of 57.4 ± 4.2 (Sperlich et al., 2011) and 55.0 ± 5.2 mL·min^−1^·kg^−1^ (Simpson et al., 2017). Therefore, polarized endurance training, as performed by middle- and long-distance runners and which includes regular high-intensity bouts (Rosenblat et al., 2019), could probably contribute to a more efficient development of endurance performance.

### Limitations

Both the results of the present study and their interpretation are limited to the publications that were available during the literature search. To be included, the publications had to meet several criteria. But at some point, particularly the methods used for CMJ determination often differed to such an extent that given variances must partly be attributed to this issue. For instance, there is no rational explanation for the fact that surfers showed a better CMJ performance than elite volleyball players, basketball players, or fencers. Sample sizes could also have had a significant impact on the results. Further, a graded exercise test with increments of 5 min duration was used to allow the determination of lactate thresholds. With regard to a previous study, this protocol might have had a small impact on the determination of VO_2_max (Wahl et al., 2018). However, the analyses show clear trends in the physical fitness of SOP operators that can be considered to design optimized training programs.

## Conclusion

In summary, SOP operators are on average heavier, taller, and stronger compared to elite athletes. But both the ability to convert this strength into explosive movement and aerobic power is significantly less developed than in most elite athletes, although SOP operators rate the importance of aerobic and muscle power as equally important as muscle strength and regularly train these physical components accordingly. Thus, the weaknesses shown here might primary be a consequence of unstructured training methods. Consequently, a frequently supervised training regimen that is aligned with those of professional athletes might lead to a more balanced development of motor abilities, in line with the occupational requirements of SOP operators described in the literature. In the future, detailed training evaluations could help further differentiate strengths and weaknesses in the fitness of SOP operators and design purposeful training interventions. Measurements of the rate of force development could improve knowledge about SOP operators' muscle power, particularly that of the upper extremities.

## Data Availability Statement

The data that support the findings of this research are available from the corresponding author upon reasonable request.

## Ethics Statement

The studies involving human participants were reviewed and approved by Ethics Committee of the German Sport University Cologne. The patients/participants provided their written informed consent to participate in this study.

## Author Contributions

J-PG, PW, and SK conceived and designed the study. LZ and MZ collected and analyzed the data. LZ drafted the manuscript. J-PG, PW, and MZ supported the correction and editing of the manuscript. All authors have read and approved the manuscript.

## Funding

This work was supported by the North Rhine-Westphalia State Police (Germany).

## Conflict of Interest

The authors declare that the research was conducted in the absence of any commercial or financial relationships that could be construed as a potential conflict of interest.

## Publisher's Note

All claims expressed in this article are solely those of the authors and do not necessarily represent those of their affiliated organizations, or those of the publisher, the editors and the reviewers. Any product that may be evaluated in this article, or claim that may be made by its manufacturer, is not guaranteed or endorsed by the publisher.
