# Supplementary material for: How Fit Are Special Operations Police Officers? A Comparison With Elite Athletes From Olympic Disciplines
Source: Front Sports Act Living. 2021 Dec 2;3:742655. doi: 10.3389/fspor.2021.742655 (PMC8674691; doi:10.3389/fspor.2021.742655)
Supplement: Supplementary file 1 [file Data_Sheet_1.PDF]

S1. Anthropometric and performance Data obtained from studies on elite athletes from Olympic disciplines. VO_2max = Maximum oxygen uptake.

Sport	Author (Year)	Sample Description	Body Mass (kg)		Body Height (cm)		VO_2max ($mL \cdot min^{-1} \cdot kg^{-1}$)			Hand Grip Strength (kg)		Countermovement Jump (cm)		
			N	Mean ± SD	N	Mean ± SD	N	Mean ± SD	Method	N	Mean ± SD	N	Mean ± SD	Method
Athletics: Sprint Running	Zinner et al. (2010)	German national sprint squad					17	55,60 ± 6,60	treadmill					
	Bong-Ju & Byoung-Goo (2017)	Elite South Korean sprinters	7	77,30 ± 5,92	7	184,30 ± 5,85	7	61,60 ± 5,20	treadmill					
	Bračič et al. (2010)	Elite Sprinters Average 100 m time: 10,82 ± 0.25 s	12	74,92 ± 5,23	12	177,58 ± 6,86						12	60,11 ± 6,35	force plate without arm swing
	Loturco et al. (2015)	Top ranked Brazilian sprinters										13	45,80 ± 5,41	force plate, without armswing
	Philpott et al. (2020)	Elite sprinters at international level	9	76,40 ± 6,40	9	181,00 ± 7,00						9	46,40 ± 6,10	force plate, without armswing
	Loturco et al. (2018)	Top-level sprinters	7	76,30 ± 10,30	7	181,60 ± 9,60						7	51,70 ± 4,90	force plate, without armswing
	**Combined**		**35**	**76,05 ± 6,65**	**35**	**180,61 ± 7,44**	**24**	**57,35 ± 6,72**				**41**	**51,13 ± 8,33**	
Athletics: Middle- and Long-Distance Running	Balsalobre-Fernández et al. (2018)	Elite middle- and long-distance runners, two intervention groups	6	69,20 ± 8,60	6	179,00 ± 7,00	6	69,10 ± 5,30	treadmill					
			6	65,20 ± 2,60	6	178,00 ± 4,00	6	72,30 ± 6,80						
	Boorsma et al. (2014)	Elite middle-distance runners	8	65,70 ± 7,00			8	80,00 ± 5,00	treadmill					
	Prommer et al. (2010)	Elite Kenyan and German middle- and long-distance runners	10	57,20 ± 7,00	10	175,70 ± 10,00	10	71,50 ± 5,00	treadmill					
			11	66,50 ± 6,30	11	180,30 ± 6,60	11	70,70 ± 3,70						
	Santos-Concejero et al. (2015a)	Elite Kenyan long-distance runners	15	54,80 ± 6,30	15	170,50 ± 6,30	15	71,90 ± 5,10	treadmill					
	Santos-Concejero et al. (2015b)	Elite Eritrean and European long-distance runners	9	57,80 ± 3,30	9	172,00 ± 5,20	9	73,50 ± 6,00	treadmill					
			8	63,50 ± 7,50	8	173,60 ± 5,10	8	77,20 ± 5,20						
	Støa et al. (2020)	Elite Norwegian long-distance runners	12	61,90 ± 11,40			12	71,20 ± 8,30	treadmill					
	Kobal et al. (2017)	Brazilian professional runners	15	59,60 ± 7,10	15	175,40 ± 2,10						15	32,37 ± 3,80	contact mat, without armswing
	Ramírez-Campillo et al. (2014)	Runners at national and international level, two intervention groups	10	63,20 ± 1,50								10	37,00 ± 5,80	contact mat, with armswing
			9	61,10 ± 3,40								9	38,10 ± 3,70	
	**Combined**		**119**	**61,38 ± 7,60**	**80**	**175,10 ± 6,70**	**85**	**72,86 ± 6,24**				**34**	**35,25 ± 5,05**	
Athletics: Marathon	Tam et al. (2012)	Kenyan and European Marathon runners	10	59,40 ± 5,80	10	172,00 ± 7,00	10	64,90 ± 5,80	treadmill					
			9	61,10 ± 4,50	9	175,00 ± 5,00	9	63,90 ± 3,70						
	Legaz-Arrese et al. (2011)	Elite Marathon runners	10	60,70 ± 3,50	10	172,00 ± 2,00	10	81,30 ± 4,00	treadmill					
	**Combined**		**29**	**60,38 ± 4,59**	**29**	**172,93 ± 5,12**	**29**	**70,24 ± 9,31**						
Athletics: High Jump	Philpott et al. (2020)	Elite high jumpers at international level	5	72,50 ± 4,40	5	182,00 ± 4,00						5	42,80 ± 4,60	force plate, without armswing
	**Combined**		**5**	**72,50 ± 4,40**	**5**	**182,00 ± 4,00**						**5**	**42,80 ± 4,60**	
Baseball	Laudner et al. (2017)	Professional baseball pitchers	66	91,30 ± 10,90	66	186,90 ± 5,70				66	59,00 ± 8,90			
	Yang (2014)	Korean professional players divided into pitchers and	46	84,40 ± 6,20	46	184,20 ± 3,50	46	53,64 ± 5,56	treadmill					
			78	82,60 ± 7,60	78	181,40 ± 4,40	78	52,30 ± 6,09						
	**Combined**		**190**	**86,06 ± 9,42**	**190**	**183,99 ± 5,26**	**124**	**52,80 ± 5,91**		**66**	**59,00 ± 8,90**			
Badminton	Heller (2010)	Czech Senior National Team					25	63,20 ± 3,70	treadmill					
	Tomaszewski et al. (2018)	Polish National Team A	9	82,70 ± 4,60	9	185,00 ± 5,10	9	55,90 ± 7,10	cycle ergometer					
		Polish National Team B	11	72,80 ± 3,70	11	183,30 ± 4,30	11	57,20 ± 3,50	cycle ergometer					
	Abián-Vicén et al. (2012)	Players participating in the Spanish national championship	46	74,54 ± 8,02	46	178,00 ± 8,00				46	46,55 ± 6,67 #	46	39,48 ± 3,63	force plate, without armswing
	Abian et al. (2015)	Elite players at national and international level	16	71,80 ± 7,90	16	174,10 ± 5,80				16	44,85 ± 7,61 #	16	37,70 ± 4,50	force plate, armswing not reported
	**Combined**		**82**	**74,67 ± 7,52**	**82**	**178,72 ± 7,57**	**45**	**60,27 ± 3,50**		**62**	**46,11 ± 6,90**	**62**	**39,02 ± 3,91**	

S1. Continued

Sport	Author (Year)	Sample Description	Body Mass (kg)		Body Height (cm)		VO_2max ($mL{\cdot}min^{-1}{\cdot}kg^{-1}$)			Hand Grip Strength (kg)		Countermovement Jump (cm)		
			N	Mean ± SD	N	Mean ± SD	N	Mean ± SD	Method	N	Mean ± SD	N	Mean ± SD	Method
Basketball	Stojanovic et al. (2012)	Elite players	24	95,70 ± 8,80	24	197,10 ± 6,20	24	51,90 ± 4,10	treadmill			24	39,80 ± 5,10	contact mat, without armswing
	Pena et al. (2018)	Spanish first division	18	92,64 ± 9,76	18	197,10 ± 5,20						18	50,14 ± 7,57	conact mat, with armswing
	Balsalobre-Fernández et al. (2014)	Spanish first division	11	98,50 ± 8,60	11	200,20 ± 10,90						11	45,60 ± 5,90	optical system, with armswing
	Tokatlidou et al. (2020)	Players divided into two groups: elite and professional	30	95,28 ± 9,86	30	198,90 ± 8,22	30	44,49 ± 5,20	treadmill			30	39,50 ± 3,33	force plate, armswing not reported
			15	95,73 ± 9,76	15	199,60 ± 7,32	15	39,54 ± 2,71				15	42,14 ± 4,61	
	Ben Abdelkrim et al. (2010)	Tunisian national team	15	91,50 ± 7,20	15	198,40 ± 6,20						15	49,70 ± 5,80	force plate, without armswing
	Shalfawi et al. (2011)	Norwegian professional players	33	89,80 ± 11,10	33	192,00 ± 8,20						33	52,00 ± 7,50	contact mat, without armswing
	**Combined**		**146**	**93,69 ± 9,84**	**146**	**196,94 ± 7,92**	**69**	**45,99 ± 6,42**				**146**	**45,47 ± 7,83**	
Boxing	Cepulenas et al. (2011)	Lithuanian National Team	10	71,87 ± 15,18	10	179,00 ± 7,70				10	53,20 ± 9,63 #	10	41,70 ± 3,00	conctact mat, with armswing
	El-Ashker & Nasr (2012)	Egyptian elite boxers	17	73,80 ± 5,10	17	175,30 ± 0,02	17	58,20 ± 6,90	treadmill					
	Bruzas et al. (2014)	Lithuanian National Team	12	79,20 ± 13,80	12	182,00 ± 9,70	12	58,00 ± 3,00						
	Halperin et al. (2016)	Professional boxer	1	80,00 ± 0,00	1	180,00 ± 0,00	1	62,30 ± 0,00	treadmill			1	40,00 ± 0,00	force plate, without armswing
	Loturco et al. (2015)	Elite armateur boxers from the Brazilian national team										9	37,42 ± 4,75	contact mat, without armswing
	**Combined**		**40**	**75,09 ± 11,26**	**40**	**178,35 ± 6,97**	**30**	**58,26 ± 5,50**		**10**	**53,20 ± 9,63**	**20**	**39,69 ± 4,28**	
Canoeing/ Kayaking	Hamano et al. (2015)	Athletes at international and national level divided into canoe paddlers and kayak paddlers	11	70,80 ± 7,80	11	172,80 ± 5,20	11	54,30 ± 4,30	treadmill	11	50,00 ± 11,50			
			12	69,50 ± 7,80	12	172,80 ± 5,30	12	55,60 ± 3,70		12	50,60 ± 7,90			
	Bielik et al. (2019)	Canoe slalom athletes from the Slovak national team					19	57,70 ± 6,80	treadmill					
	Peeling et al. (2015)	Kayakers at national level	6	87,40 ± 7,50	6	182,50 ± 8,10	6	57,15 ± 2,77	kayak ergometer					
	García-Pallarés et al. (2010)	Elite flat-water kayakers	10	85,30 ± 5,60	10	184,00 ± 5,90	10	65,39 ± 3,88	kayak ergometer					
	Zouhal et al. (2012)	Kayakers at national level	7	78,54 ± 3,41	7	184,00 ± 3,00	7	68,00 ± 8,00	graded test on water, portable gas analyzer					
	**Combined**		**46**	**76,96 ± 9,85**	**46**	**178,20 ± 7,64**	**65**	**58,98 ± 7,00**		**23**	**50,31 ± 9,56**			
Cycling BMX	Louis et al. (2013)	Elite BMX cyclists at international level	6	75,40 ± 3,30	6	177,30 ± 9,70	6	54,70 ± 4,70	cycle ergometer					
	Zabala et al. (2011)	Elite BMX cyclists	10	77,90 ± 2,10	10	178,30 ± 2,10						10	32,15 ± 2,33	contact mat, armswing not reported
	**Combined**		**16**	**76,96 ± 2,80**	**16**	**177,93 ± 5,85**	**6**	**54,70 ± 4,70**				**10**	**32,15 ± 2,33**	
Cycling XC MTB	Bejder et al. (2019)	Elite Danish athletes at national and itnernational level	11	70,20 ± 7,20	11	182,00 ± 6,00	11	71,10 ± 7,40	cycle ergometer					
	Macdermid & Stannard (2012)	Elite cyclists at national level	7	66,90 ± 7,70	7	176,00 ± 4,00	7	67,60 ± 5,30	cycle ergometer					
	**Combined**		**18**	**68,92 ± 7,36**	**18**	**179,67 ± 5,99**	**18**	**69,74 ± 6,72**						
Cycling Road	Rønnestad et al. (2020)	Cyclists at national level divided into two intervention groups	9	75,20 ± 3,60	9	181,00 ± 4,00	9	73,30 ± 3,60	cycle ergometer					
			9	74,90 ± 6,10	9	183,00 ± 4,00	9	72,70 ± 4,90						

S1. Continued

Sport	Author (Year)	Sample Description	Body Mass (kg)		Body Height (cm)		VO_2max ($mL \cdot min^{-1} \cdot kg^{-1}$)			Hand Grip Strength (kg)		Countermovement Jump (cm)		
			N	Mean ± SD	N	Mean ± SD	N	Mean ± SD	Method	N	Mean ± SD	N	Mean ± SD	Method
Cycling Road	Rønnestad et al. (2017)	Cyclists at national level divided into two intervention groups	12	66,50 ± 8,30	12	178,00 ± 9,00	12	77,00 ± 6,00	cycle ergometer					
			8	72,10 ± 9,40	8	181,00 ± 10,00	8	72,00 ± 7,00						
	Moro et al. (2020)	Elite Italian cyclists	16	69,66 ± 6,11			8	71,98 ± 3,93	cycle ergometer					
							8	68,30 ± 7,19						
	Bouillod & Grappe (2017)	Cyclists licensed to the French Cycling Federation	13	66,20 ± 5,60	13	177,80 ± 4,70	13	79,80 ± 6,90	cycle ergometer					
	Czuba et al. (2011)	Elite cyclists at national and international level divided into two intervention groups	10	66,70 ± 5,40	10	178,00 ± 5,00	10	67,80 ± 2,50	cycle ergometer					
			10	69,20 ± 5,50	10	179,00 ± 3,00	10	67,70 ± 2,00						
	Menaspà et al. (2015)	Competitive cyclists at international level	10	69,50 ± 4,90	10	181,00 ± 7,00	10	72,50 ± 4,40	cycle ergometer					
	**Combined**		**97**	**69,64 ± 6,77**	**81**	**179,65 ± 6,24**	**97**	**72,72 ± 6,35**						
Fencing	Kozłowska et al. (2020)	Elite fencers	10	85,50 ± 11,60			10	46,00 ± 7,80	bicycle ergometer					
	Ghloum & Hajji (2011)	Kuwaiti national fencing team	15	71,10 ± 15,00	15	175,20 ± 6,10	15	49,60 ± 4,76	treadmill					
	Tsolakis & Bogdanis (2012)	Greek national team	10	77,10 ± 6,96	10	180,00 ± 6,32						10	38,20 ± 1,90	contact mat, without armswing
	Abdollah et al. (2014)	Fencers from the Iranian national team	24	78,00 ± 8,00	24	181,00 ± 6,00	24	47,00 ± 5,30	treadmill	24	57,70 ± 8,10			
	**Combined**		**59**	**77,36 ± 11,39**	**49**	**179,02 ± 6,51**	**49**	**47,59 ± 5,78**		**24**	**57,70 ± 8,10**	**10**	**38,20 ± 1,90**	
Field Hockey	Hinrichs et al. (2010)	German National Team	17	80,70 ± 7,50	17	181,00 ± 6,00	17	55,80 ± 4,00	treadmill					
	Krzykala et al. (2018)	Polish Youth National Team (21.4 ± 1.6 years)	15	75,30 ± 9,00	15	178,00 ± 5,00				15	51,33 ± 4,89			
	Bartolomei et al. (2019)	Players from the Italian first division	15	71,60 ± 8,50	15	174,60 ± 6,50				15	49,75 ± 4,76			
	**Combined**		**47**	**76,07 ± 8,99**	**47**	**178,00 ± 6,33**	**17**	**55,80 ± 4,00**		**30**	**50,54 ± 4,81**			
Football	Bekris et al. (2019)	Greek Super League	24	77,40 ± 6,10	24	180,30 ± 3,80	24	56,50 ± 3,50	treadmill					
	Fessi et al. (2016)	Tunisian Stars League	22	72,90 ± 8,40	22	177,90 ± 6,00						22	47,70 ± 4,90	force plate, with armswing
	Krommes et al. (2017)	Danish 1st Division, two intervention groups	9	73,10 ± 5,80	9	183,00 ± 5,00						9	43,80 ± 3,70	force plate, without armswing
			10	77,90 ± 9,90	10	181,00 ± 7,00						10	42,60 ± 6,70	
	Meckel et al. (2018)	Israeli 1st Division	18	76,90 ± 8,40		±	18	55,60 ± 6,30	treadmill			18	38,80 ± 5,30	force plate, with armswing
	Requena et al. (2017)	Spanish La Liga	19	76,00 ± 6,90	19	180,30 ± 3,30						19	51,10 ± 5,70	contact mat without armswing
	Nilsson & Cardinale (2015)	Players from the Swedish first division	39	79,00 ± 7,60	39	180,00 ± 5,90	39	57,00 ± 4,75	treadmill			39	48,90 ± 4,40	optical system, with armswing
	Boone et al. (2012)	Belgian first divison players	289	77,40 ± 7,10	289	182,40 ± 6,00	289	57,70 ± 4,70	treadmill			289	43,10 ± 4,90	contact mat without armswing
	Helgerud et al. (2011)	Player from the Norwegian Premier League	21	78,40 ± 7,41	21	184,00 ± 5,42	21	60,50 ± 4,78	treadmill			21	57,17 ± 4,84	force plate, without armswing
	Kobal et al. (2017)	Brazilian top-level players	21	77,60 ± 7,00	21	180,30 ± 6,20						21	40,95 ± 2,95	contact mat without armswing
	Randell et al. (2019)	Spanish La Liga (FC Barcelona)	16	74,30 ± 6,50	16	180,20 ± 7,10	16	57,10 ± 3,80	treadmill					
	**Combined**		**488**	**77,13 ± 7,29**	**470**	**181,68 ± 5,95**	**407**	**57,59 ± 4,75**				**448**	**44,56 ± 6,17**	
Golf	Saiful Annur et al. (2017)	Elite golfers (handicap < 5)	8	74,30 ± 12,80	8	174,00 ± 2,00				8	44,00 ± 4,46 #			
	Álvarez et al. (2012)	Spanish golfers (handicap < 5) divided into two intervention groups	5	70,76 ± 7,10	5	177,22 ± 3,00				5	40,45 ± 7,01 #	5	31,70 ± 4,29	contact mat, without armswing
			5	68,09 ± 8,30	5	171,62 ± 6,00				5	45,64 ± 8,40 #	5	35,55 ± 1,66	
	Parker et al. (2017)	Golfers at national and international level	13	76,80 ± 11,00	13	178,70 ± 7,30						13	43,70 ± 7,10	optical system, with armswing
	**Combined**		**31**	**73,78 ± 10,65**	**31**	**176,11 ± 5,99**				**18**	**43,47 ± 6,37**	**23**	**39,32 ± 7,69**	

S1. Continued

Sport	Author (Year)	Sample Description	Body Mass (kg) N	Body Mass (kg) Mean ± SD	Body Height (cm) N	Body Height (cm) Mean ± SD	VO_2max ($mL \cdot min^{-1} \cdot kg^{-1}$) N	VO_2max Mean ± SD	VO_2max Method	Hand Grip Strength (kg) N	Hand Grip Strength (kg) Mean ± SD	Countermovement Jump (cm) N	Countermovement Jump (cm) Mean ± SD	Countermovement Jump Method
Gymnastics Artistic	Dallas et al. (2013)	Elite Greek gymnasts	11	58,09 ± 8,21	11	161,18 ± 6,96	11	50,60 ± 4,80	treadmill					
	Donti et al. (2014)	Greek national team	10	64,30 ± 13,91	10	167,00 ± 9,49						10	38,50 ± 2,85	contact mat, without armswing
	Paoli et al. (2012)	Elite gymnasts from the Italian premier league	9	69,60 ± 7,30								9	45,00 ± 4,00	contact mat, without armswing
	Sterkowicz-Przybycień et al. (2019)	Gymnasts competing at international and national level	19	68,30 ± 7,90	19	170,00 ± 4,50				19	46,60 ± 9,00	19	39,30 ± 4,59	optical system, without armswing
	**Combined**		**49**	**65,43 ± 10,09**	**40**	**166,82 ± 7,51**	**11**	**50,60 ± 4,80**		**19**	**46,60 ± 9,00**	**38**	**40,44 ± 4,74**	
Handball	Pena et al. (2018)	Spanish first division	15	94,01 ± 8,89	15	191,03 ± 5,66						15	48,76 ± 4,53	conact mat, with armswing
	Michalsik et al. (2015)	Danish Premier Team Handball League	26	90,90 ± 9,00	26	188,90 ± 6,30	26	57,00 ± 4,10	treadmill			26	43,90 ± 6,00	contact mat, without armswing
	Dello Iacono et al. (2017)	Elite players, two intervention groups	18	87,80 ± 7,40	18	192,50 ± 3,70						9	42,70 ± 5,60	force plate without armswing
												9	41,50 ± 3,80	
	Wagner et al. (2019)	First Austrian Team Handball League	12	90,00 ± 11,00	12	188,00 ± 6,00	12	52,00 ± 4,20	treadmill			12	40,00 ± 5,60	force plate without armswing
	Hermassi et al. (2014)	Tunisian players at the highest national level, two intervention groups	24	89,10 ± 2,10	24	188,00 ± 7,00						14	44,28 ± 0,60	force plate without armswing
												10	41,68 ± 0,95	
	Lijewski et al. (2019)	Polish professionals divded into 4 groups: goalkeepers, wingers, backs, pivot players	5	98,20 ± 14,20	5	187,60 ± 5,90				5	60,70 ± 2,45			
			7	81,90 ± 7,20	7	181,10 ± 4,70				7	51,80 ± 6,25			
			14	93,70 ± 6,80	14	187,70 ± 6,10				14	58,60 ± 7,45			
			6	104,50 ± 10,90	6	193,40 ± 7,80				6	59,10 ± 7,40			
	González-Ravé et al. (2014)	Players from Spanish first division	12	92,89 ± 12,34	12	190,00 ± 5,00						12	35,89 ± 4,20	force plate, without armswing
	**Combined**		**139**	**91,30 ± 9,31**	**139**	**189,09 ± 6,28**	**38**	**55,42 ± 4,71**		**32**	**57,53 ± 7,10**	**107**	**42,79 ± 5,66**	
Judo	Drid et al. (2015)	Elite and sub-elite European judokas	5	100,30 ± 2,17	5	188,90 ± 7,67	5	55,99 ± 2,24	treadmill	5	66,65 ± 6,76 #			
			5	100,70 ± 1,86	5	185,80 ± 4,29	5	48,72 ± 1,72		5	60,40 ± 5,70 #			
	Silva et al. (2011)	National top-level judoka	27	72,80 ± 7,10	27	176,00 ± 5,00				27	50,80 ± 7,40			
	Zaggelidis (2016)	Elite judoka at national and international level	16	84,24 ± 12,38	16	181,00 ± 2,00				16	72,53 ± 7,94 #			
	**Combined**		**53**	**81,48 ± 13,46**	**53**	**179,65 ± 6,24**	**10**	**52,36 ± 4,27**		**53**	**59,76 ± 12,17**			
Karate	Koropanovski et al. (2011)	Members of the Serbian national karate team, two groups: Kumite, Kata	19	77,60 ± 10,90	19	181,30 ± 8,00						19	46,10 ± 4,40	contact mat, with armswing
			12	70,50 ± 5,00	12	174,30 ± 5,50						12	48,66 ± 8,10	
	Loturco et al. (2014)	Professionals from the Brazilian national team	9	76,70 ± 14,40								9	43,20 ± 5,30	contact mat, without armswing
	Zaggelidis (2016)	Elite kumite karatekas at national and international level	18	76,63 ± 10,46	18	182,00 ± 8,00				18	65,78 ± 7,72 #			
	Najmi et al.(2018)	Elite Malaysian athletes at national and international level	8	66,27 ± 10,50	8	173,11 ± 7,29				8	47,41 ± 6,12	8	37,87 ± 2,47	force plate, without armswing
	**Combined**		**66**	**74,55 ± 10,92**	**57**	**178,90 ± 8,18**				**26**	**60,13 ± 11,21**	**48**	**44,82 ± 6,45**	
Modern Pentathlon	Lim et al. (2018)	Elite South Korean athletes at international level	7	69,50 ± 3,39	7	177,00 ± 4,56	7	72,05 ± 6,58	treadmill					
	**Combined**		**7**	**69,50 ± 3,39**	**7**	**177,00 ± 4,56**	**7**	**72,05 ± 6,58**						
Rowing	Klusiewicz et al. (2014)	Top Polish rowing crews: quadruple scull, lightweight coxless four 1, lightweight coxless four 2					4	64,90 ± 6,10	rowing ergometer					
							4	70,70 ± 7,10						
							4	71,90 ± 7,30						
	Mikulic (2011)	Elite Croatian rowers	4	95,00 ± 4,35	4	188,00 ± 1,82	4	69,70 ± 5,50	rowing ergometer					

S1. Continued

Sport	Author (Year)	Sample Description	Body Mass (kg) N	Body Mass (kg) Mean ± SD	Body Height (cm) N	Body Height (cm) Mean ± SD	VO_2max ($mL \cdot min^{-1} \cdot kg^{-1}$) N	VO_2max Mean ± SD	VO_2max Method	Hand Grip Strength (kg) N	Hand Grip Strength (kg) Mean ± SD	Countermovement Jump (cm) N	Countermovement Jump (cm) Mean ± SD	Countermovement Jump Method
Rowing	Nevill et al. (2011)	Current or former elite rowers, two groups: heavy and light weight	33	94,70 ± 5,90	33	192,40 ± 5,40	33	61,70 ± 4,80	rowing ergometer					
			15	74,50 ± 2,80	15	181,30 ± 4,10	15	68,20 ± 5,37						
	Das et al. (2019)	Elite Indian rowers at international level, light weight and open category	15	70,72 ± 1,20	15	182,73 ± 3,59				15	51,13 ± 3,85			
			12	79,13 ± 2,54	12	185,58 ± 3,37				12	56,21 ± 4,54			
	Lindenthaler et al. (2018)	International class rowers	22	88,90 ± 9,80	22	190,80 ± 7,30	22	66,30 ± 3,60	rowing ergometer					
	**Combined**		**101**	**85,04 ± 11,11**	**101**	**187,98 ± 6,76**	**86**	**65,42 ± 5,81**		**27**	**53,39 ± 4,83**			
Rugby	Comfort et al. (2011)	English Super League players	18	96,87 ± 10,92	18	184,16 ± 5,76						18	38,50 ± 4,90	contact mat, without armswing
	Ireton et al. (2019)	English Super League players	18	87,00 ± 8,80	18	184,90 ± 7,90						18	34,00 ± 11,00	force plate, without armswing
	La Monica et al. (2016)	Collegiate rugby player, two groups: forwards, backs	13	90,50 ± 12,40	13	180,00 ± 10,00	9	49,40 ± 4,70	treadmill					
			12	73,70 ± 7,10	12	180,00 ± 10,00	9	54,90 ± 3,90						
	Clarke et al. (2019)	English Super League players	8	94,90 ± 11,40	8	189,20 ± 7,20						8	48,9 ± 3,9	contact mat, armswing not reported
	Crewther et al. (2012)	Professional rugby union team players	16	115,60 ± 12,40								16	39,6 ± 8,6	force plate, without armswing
			16	103,50 ± 8,90								16	41,8 ± 4,9	
			16	97,60 ± 12,50								16	39,7 ± 5,9	
			16	101,60 ± 15,20								16	34,2 ± 5,7	
	**Combined**		**133**	**96,41 ± 15,41**	**69**	**183,43 ± 8,49**	**18**	**52,15 ± 5,06**				**108**	**38,71 ± 7,96**	
Sailing	Bay et al. (2018)	Elite Sailors from the Danish national team (49er class), two groups	9	79,00 ± 7,00	9	185,00 ± 8,00	9	56,30 ± 4,70	treadmill					
			6	82,00 ± 2,00	6	184,00 ± 8,00	6	58,50 ± 3,90						
	Philippe et al. (2020)	Professional inshore sailors at international level	21	74,78 ± 6,56	21	179,32 ± 7,30	21	53,40 ± 8,50	treadmill	21	59,05 ± 4,75 #	21	38,60 ± 5,60	conact mat, without armswing
	**Combined**		**36**	**77,04 ± 6,68**	**36**	**181,52 ± 7,83**	**36**	**54,98 ± 7,25**		**21**	**59,05 ± 4,75**	**21**	**38,60 ± 5,60**	
Shooting	Mon et al. (2015)	Pistol shooters of the Spanish Championship	46	87,38 ± 13,30	46	175,00 ± 7,00				46	47,92 ± 7,26			
	**Combined**		**46**	**87,38 ± 13,30**	**46**	**175,00 ± 7,00**				**46**	**47,92 ± 7,26**			
Sport Climbing	Limonta et al. (2018)	French climbers at national level divided into two groups: advanced, elite	7	68,80 ± 6,00	7	179,00 ± 7,00	7	53,11 ± 2,65	cycle ergometer					
			6	67,20 ± 4,30	6	177,00 ± 7,00	6	54,06 ± 6,56						
	Macdonald & Callender (2011)	Highly accomplished boulderers	12	70,20 ± 6,20	12	177,70 ± 4,90				12	57,31 ± 7,04			
	Limonta et al. (2016)	Elite climbers competing at international level	11	63,20 ± 2,90	11	171,00 ± 4,00				11	77,70 ± 3,47			
	Callender et al. (2021)	Elite climbers (Grade Fontainebleau 8b)					7	53,00 ± 4,10	treadmill					
	**Combined**		**36**	**67,29 ± 5,65**	**36**	**175,79 ± 6,19**	**20**	**53,36 ± 4,37**		**23**	**67,06 ± 11,78**			
Surfing	Secomb et al. (2016)	Surfers at internaitonal level	10	75,00 ± 4,90	10	178,00 ± 4,00						10	51,00 ± 4,00	force plate, without armswing
	Furness et al. (2018)	Surfers competing on the WQS or WCT	15	77,83 ± 6,62	15	179,44 ± 3,96	15	40,71 ± 3,28	swim bench ergometer					
	Almeida et al. (2018)	Elite surfers at international level	6	68,50 ± 3,80	6	174,00 ± 5,00	6	43,70 ± 7,90	swim bench ergometer					
	**Combined**		**31**	**75,11 ± 6,52**	**31**	**177,92 ± 4,53**	**21**	**41,56 ± 5,00**				**10**	**51,00 ± 4,00**	
Swimming	Acar & Eler (2018)	Elite zweifelhaft	41	81,71 ± 5,58	41	184,48 ± 5,39				41	46,73 ± 6,19 #			
	Garrido et al. (2012)	Portuguese swimmers at national level	18	76,24 ± 6,13	18	181,67 ± 5,92				18	50,08 ± 7,01 #			
	Buśko & Gajeski (2011)	Elite swimmers at international level	8	82,50 ± 3,80	8	188,80 ± 6,00						8	42,00 ± 5,00	force plate, with arm swing

S1. Continued

Sport	Author (Year)	Sample Description	Body Mass (kg)		Body Height (cm)		VO_2max (mL·min^{-1}·kg^{-1})			Hand Grip Strength (kg)		Countermovement Jump (cm)		
			N	Mean ± SD	N	Mean ± SD	N	Mean ± SD	Method	N	Mean ± SD	N	Mean ± SD	Method
Swimming	Rodriguez et al. (2015)	Elite swimmers at international level divided into four intervention groups	3	76,70 ± 9,50	3	185,70 ± 5,50	3	52,40 ± 2,70	incremental swimming test, portable gas analyzer					
			7	79,20 ± 6,80	7	186,30 ± 4,90	7	53,30 ± 6,50						
			6	80,00 ± 7,10	6	186,00 ± 6,40	6	57,80 ± 7,40						
			8	81,80 ± 7,80	8	185,00 ± 5,60	8	51,70 ± 9,10						
	Chaverri et al. (2016)	Elite swimmers of national or Olympic teams	13	82,10 ± 7,20	13	187,90 ± 6,00	13	52,33 ± 5,22	200 m all out, portable gas analyzer					
	Knab et al. (2013)	Elite swimmers at national level					9	53,10 ± 1,40	cycle ergometer					
	Mejias et al. (2014)	Masters and elite swimmers at international level	8	79,63 ± 8,60	8	178,00 ± 4,00	8	62,98 ± 4,63	incremental swimming test, backward extrapolation					
			12	72,83 ± 6,44	12	179,00 ± 6,00	12	73,65 ± 10,93						
	West et al. (2011)	International sprint swimmers	11	78,10 ± 11,20	11	180,00 ± 1,00						11	34,70 ± 4,60	force plate, without armswing
	Dalamitros et al. (2019)	Swimmers within a top 8 national ranking	11	77,60 ± 6,60	11	179,70 ± 6,90						11	37,70 ± 5,30	optical system, without armswing
	**Combined**		**146**	**79,40 ± 7,25**	**146**	**183,37 ± 6,18**	**66**	**58,13 ± 10,57**		**59**	**47,75 ± 6,58**	**30**	**37,75 ± 5,62**	
Table Tennis	Zagatto et al. (2014)	National level table tennis players	11	70,80 ± 3,90			11	43,90 ± 1,40	treadmill					
	**Combined**		**11**	**70,80 ± 3,90**			**11**	**43,90 ± 1,40**						
Taekwondo	Batra & Zatoń (2016)	Taekwondo practitioners at national and international level, two intervention groups	10	73,10 ± 9,26	10	178,00 ± 7,02	10	50,13 ± 3,81	treadmill					
			10	71,63 ± 10,97	10	177,60 ± 7,35	10	53,45 ± 2,06						
	Chiodo et al. (2011)	Elite Italian Taekwondo athletes	11	78,60 ± 14,00			11	63,20 ± 6,10	treadmill	11	49,56 ± 8,97	11	40,80 ± 4,90	optical system, without armswing
	Ghorbanzadeh et al. (2011)	Athletes from the Turkish national team	24	71,12 ± 10,69	24	181,00 ± 7,07				24	55,20 ± 13,32			
	Khayyat et al. (2020)	Athletes from the Turkish national team	12	72,90 ± 6,90	12	182,00 ± 4,00	12	54,10 ± 4,40	treadmill			12	43,50 ± 6,10	force plate, armswing not reported
	**Combined**		**67**	**73,04 ± 10,60**	**56**	**180,07 ± 6,64**	**43**	**55,35 ± 6,47**		**35**	**53,43 ± 12,28**	**23**	**42,21 ± 5,61**	
Tennis	Baiget et al. (2016)	players at international and national level	8	70,80 ± 5,50	8	180,10 ± 7,30	8	60,60 ± 5,10	field test with portable gas analyzer					
			30	73,20 ± 9,40	30	180,10 ± 8,30	30	55,70 ± 5,40						
	Kobal et al. (2017)	Brazilian top-level players	24	72,40 ± 7,80	24	177,40 ± 7,00						24	39,14 ± 4,27	contact mat without armswing
	Fernandez-Fernandez et al. (2015)	Elite players (ATP ranking between 500-800)	12	76,40 ± 5,90	12	182,00 ± 22,00						12	41,10 ± 2,40	contact mat without armswing
	Bonato et al. (2015)	Elite players (ATP ranking between 300-800)	8	79,70 ± 4,30	8	181,00 ± 4,00				8	52,40 ± 6,10	8	42,70 ± 8,00	force plate, with armswing
	Thiel et al. (2011)	Professional players with ATP tour experience					3	58,67 ± 8,74	treadmill					
	**Combined**		**82**	**73,83 ± 7,99**	**82**	**179,68 ± 10,62**	**41**	**56,87 ± 5,79**		**8**	**52,40 ± 6,10**	**44**	**40,32 ± 4,86**	
Triathlon	González-Parra et al. (2013)	Elite triathletes	4	66,73 ± 6,52			4	76,00 ± 6,90	treadmill					
	McKay et al. (2020)	Elite triathletes	4	64,30 ± 4,10			4	76,60 ± 1,60	treadmill					
	Hough et al. (2015)	Elite triathletes	7	70,40 ± 3,80	7	178,00 ± 6,00	7	67,60 ± 4,50	cycle ergometer					
	Hoffmann et al. (2017)	Elite German triathletes	11	74,46 ± 4,28	11	187,00 ± 2,90	11	72,02 ± 4,29	treadmill					
	**Combined**		**26**	**70,61 ± 5,76**	**18**	**183,50 ± 6,17**	**26**	**72,15 ± 5,44**						
Volleyball	Pena et al. (2018)	Spanish first division	13	88,49 ± 7,37	13	193,23 ± 5,90						13	58,59 ± 4,62	conact mat, with armswing
												13	46,90 ± 4,10	conact mat, without armswing

S1. Continued

Sport	Author (Year)	Sample Description	Body Mass (kg)		Body Height (cm)		VO_2max ($mL \cdot min^{-1} \cdot kg^{-1}$)			Hand Grip Strength (kg)		Countermovement Jump (cm)		
			N	Mean ± SD	N	Mean ± SD	N	Mean ± SD	Method	N	Mean ± SD	N	Mean ± SD	Method
Volleyball	Manna et al. (2011)	Elite Indian volleyball players	30	81,60 ± 4,60	30	189,40 ± 4,20	30	49,80 ± 4,60	treadmill	30	41,60 ± 3,17 #			
	Đurković et al. (2014)	Croatian players with national team status	34	87,24 ± 9,98	34	192,22 ± 7,10	34	55,60 ± 4,70	treadmill					
	Horta et al. (2017)	Brazilian Volleyball Super League players	12	94,90 ± 11,60	12	194,60 ± 8,00						12	46,60 ± 5,80	contact mat, without armswing
	Borràs et al. (2011)	Spanish national Volleyball team	15	87,80 ± 9,90	15	193,30 ± 8,20						15	56,80 ± 6,40	conact mat, with armswing
	Berriel et al. (2020)	Brazilian national team	13	91,50 ± 8,80	13	193,10 ± 6,40						13	49,83 ± 7,00	conact mat, with armswing
	Kitamura et al. (2017)	Brazilian first division	9	90,20 ± 12,40	9	194,90 ± 11,00						9	49,26 ± 6,88	force plate, with armswing
	Giatsis et al. (2018)	15 elite players at international level	15	83,40 ± 6,00	15	187,00 ± 5,00						15	35,30 ± 6,10	force plate, with armswing
	**Combined**		**141**	**87,04 ± 9,37**	**141**	**191,73 ± 6,99**	**64**	**52,88 ± 5,46**		**30**	**41,60 ± 3,17**	**90**	**48,92 ± 9,45**	
Water Polo	Ramos Veliz et al. (2014)	Elite players at national level, two intervention groups	27	81,43 ± 8,48	27	180,33 ± 5,90						11	34,48 ± 4,80	optical system, armswing not reported
												16	32,38 ± 6,50	
	Galy et al. (2014)	Players from national teams	8	85,50 ± 11,70	8	180,20 ± 8,60	8	57,40 ± 5,60	treadmill					
	Ferragut et al. (2011)	Spanish water polo team	13	91,53 ± 11,96	13	188,19 ± 6,11				13	44,24 ± 6,64			
	Ferragut et al. (2015)	Players from the Spanish King's Cup	94	86,30 ± 10,70	94	185,90 ± 10,70				94	56,19 ± 6,42			
	Saez de Villarreal et al. (2015)	Players from the Spanish first division divided into three intervention groups	30	77,55 ± 5,40	30	182,60 ± 4,30						10	38,50 ± 4,00	optical system, armswing not reported
												10	39,10 ± 3,10	
												10	39,30 ± 3,10	
	**Combined**		**172**	**84,37 ± 10,46**	**172**	**184,36 ± 9,09**	**8**	**57,40 ± 5,60**		**107**	**54,74 ± 7,52**	**57**	**36,25 ± 5,48**	
Weightlifting	Erdağı (2020)	Elite Olympic style weightlifters								25	58,80 ± 14,38 #			
	**Combined**									**25**	**58,80 ± 14,38**			
Wrestling	García-Pallarés et al. (2011)	Elite wrestlers, three classes: light, middle, heavy weight	18	60,90 ± 4,40	18	167,20 ± 4,60				18	44,95 ± 6,66 #	18	35,40 ± 6,70	contact mat, without armswing
			18	73,10 ± 4,80	18	173,90 ± 5,20				18	51,10 ± 8,44 #	18	35,00 ± 3,50	
			10	87,00 ± 4,30	10	175,50 ± 5,00				10	55,75 ± 7,77 #	10	35,50 ± 4,40	
	Morán-Navarro et al. (2015)	Elite wrestlers	28	70,60 ± 12,70	28	170,80 ± 6,00	28	54,00 ± 8,20	treadmill					
	Nikooie et al. (2017)	Elite wrestlers, two intervention groups	5	87,20 ± 23,80	5	175,00 ± 9,00				5	59,00 ± 4,00			
			7	97,20 ± 22,40	7	182,00 ± 8,00				7	52,00 ± 5,90			
	Khanbabazadeh et al. (2016)	Iranian world class wrestlers	13	80,92 ± 21,06	13	176,00 ± 10,00				13	62,85 ± 7,61			
	**Combined**		**99**	**75,02 ± 16,32**	**99**	**172,87 ± 7,45**	**28**	**54,00 ± 8,20**		**71**	**52,99 ± 9,44**	**46**	**35,27 ± 5,05**	

Grip strength of the left and right hand were retrospectively averaged.

REFERENCE LIST

Abdollah, S., Khosrow, E., & Sajad, A. (2014). Comparison of Anthropometric and Functional Characteristics of Elite Male Iranian Fencers in Three Weapons. *International Journal of Applied Sports Sciences, 26*(1), 11-17.

Acar, H., & Eler, N. (2018). The Relationship of Digit Ratio (2D:4D) With Cerebral Lateralization and

Grip Strength in Elite Swimmers. *Journal of education and training studies, 6*(4), 84-89.

Almeida, N., Reis, J., Beckert, J., Moreira, M., & Alves, F. (2018). Peak oxygen uptake differentiates competitive from recreational male surfboard riders. *Motricidade, 13*(4), 39. doi:10.6063/motricidade.11730

Alvarez, M., Sedano, S., Cuadrado, G., & Redondo, J. C. (2012). Effects of an 18-week strength training program on low-handicap golfers' performance. *Journal of Strength and Conditioning Research, 26*(4), 1110-1121. doi:10.1519/JSC.0b013e31822dfa7d

Baiget, E., Iglesias, X., & Rodríguez, F. (2016). Aerobic Fitness and Technical Efficiency at High Intensity Discriminate between Elite and Subelite Tennis Players. *International Journal of Sports Medicine, 37*(11), 848-854. doi:10.1055/s-0042-104201

Balsalobre-Fernández, C., Romero-Moraleda, B., Cupeiro, R., Peinado, A. B., Butragueño, J., & Benito, P. J. (2018). The effects of beetroot juice supplementation on exercise economy, rating of perceived exertion and running mechanics in elite distance runners: A double-blinded, randomized study. *PloS One, 13*(7), e0200517. doi:10.1371/journal.pone.0200517

Balsalobre-Fernández, C., Tejero-González, C. M., del Campo-Vecino, J., & Bachero-Mena, B. (2014). Relationships among repeated sprint ability, vertical jump performance and upper-body strength in professional basketball players. *Arch Med Deporte, 31*(3), 148-153.

Bartolomei, S., Nigro, F., Gubellini, L., Ciacci, S., Merni, F., Treno, F., . . . Semprini, G. (2019). Physiological and Sport-Specific Comparison Between Division I and Division II Italian Male Field Hockey Players. *Journal of Strength and Conditioning Research, 33*(11), 3123-3128. doi:10.1519/JSC.0000000000002503

Batra, A., & Zatoń, M. (2016). Effect of high intensity interval training on cardiopulmonary function in Taekwon-do ITF athletes. *Journal of Combat Sports and Martial Arts, 7*(1), 73-79. doi:10.5604/20815735.1225636

Bay, J., Bojsen-Moller, J., & Nordsborg, N. B. (2018). Reliable and sensitive physical testing of elite trapeze sailors. *Scand J Med Sci Sports, 28*(3), 919-927. doi:10.1111/sms.12993

Bejder, J., Bonne, T. C., Nyberg, M., Sjøberg, K. A., & Nordsborg, N. B. (2019). Physiological determinants of elite mountain bike cross-country Olympic performance. *Journal of Sports Sciences, 37*(10), 1154-1161. doi:10.1080/02640414.2018.1546546

Bekris, E., Mylonis, E., Gissis, I., Katis, A., Metaxas, T., Komsis, S., & Kompodieta, N. (2019). Variation of aerobic performance indices of professional elite soccer players during the annual macrocycle. *Journal of Sports Medicine and Physical Fi ness, 59*(10), 1628-1634. doi:10.23736/S0022-4707.19.09800-1

Ben Abdelkrim, N., Chaouachi, A., Chamari, K., Chtara, M., & Castagna, C. (2010). Positional role and competitive-level differences in elite-level men's basketball players. *Journal of Strength and Conditioning Research, 24*(5), 1346-1355. doi:10.1519/JSC.0b013e3181cf7510

Berriel, G. P., Schons, P., Costa, R. R., Oses, V. H. S., Fischer, G., Pantoja, P. D., . . . Peyre-Tartaruga, L. A. (2020). Correlations Between Jump Performance in Block and Attack and the Performance in Official Games, Squat Jumps, and Countermovement Jumps of Professional Volleyball Players. *Journal of Strength and Conditioning Research, Publish Ahead of Print*. doi:10.1519/JSC.0000000000003858

Bielik, V., Dalcheco Messias, L. H., Vajda, M., Lopata, P., Chudý, J., & Manchado-Gobatto, F. D. B. (2019). Is the aerobic power a delimitating factor for performance on canoe slalom? An analysis of Olympic Slovak canoe slalom medalists and non-Olympics since Beijing 2008 to Rio 2016. *Journal of Human Sport and Exercise, 14*(4). doi:10.14198/jhse.2019.144.16

Bonato, M., Maggioni, M. A., Rossi, C., Rampichini, S., La Torre, A., & Merati, G. (2015). Relationship between anthropometric or functional characteristics and maximal serve velocity in professional tennis players. *Journal of Sports Medicine and Physical Fi ness, 55*(10), 1157-1165. Retrieved from https://www.ncbi.nlm.nih.gov/pubmed/24998615

Bong-Ju, S., & Byoung-Goo, K. (2017). Differences of Physique and Physical Fitness among Male South Korean Elite National Track and Field Athletes. *International Journal of Human Movement and Sports Sciences, 5*(2), 17-26. doi:10.13189/saj.2017.050201

Boone, J., Vaeyens, R., Steyaert, A., Vanden Bossche, L., & Bourgois, J. (2012). Physical fitness of elite Belgian soccer players by player position. *Journal of Strength and Conditioning Research, 26*(8), 2051-2057. doi:10.1519/JSC.0b013e318239f84f

Boorsma, R. K., Whitfield, J., & Spriet, L. L. (2014). Beetroot juice supplementation does not improve performance of elite 1500-m runners. *Medicine and Science in Sports and Exercise, 46*(12), 2326-2334. doi:10.1249/MSS.0000000000000364

Borras, X., Balius, X., Drobnic, F., & Galilea, P. (2011). Vertical jump assessment on volleyball: a follow-up of three seasons of a high-level volleyball team. *Journal of Strength and Conditioning Research, 25*(6), 1686-1694. doi:10.1519/JSC.0b013e3181db9f2e

Bouillod, A., & Grappe, F. (2018). Physiological and biomechanical responses between seated and standing positions during distancebased uphill time trials in elite cyclists. *Journal of Sports Sciences, 36*(10), 1173-1178. doi:10.1080/02640414.2017.1363902

Bračič, M., Supej, M., Peharec, S., Bačić, P., & Čoh, M. (2010). AN INVESTIGATION OF THE INFLUENCE OF BILATERAL DEFICIT ON THE COUNTER-MOVEMENT JUMP PERFORMANCE IN ELITE SPRINTERS. *Kinesiology, 42*(1), 73-81.

Bruzas, V., Stasiulis, A., Cepulenas, A., Mockus, P., Statkeviciene, B., & Subacius, V. (2014). Aerobic Capacity is Correlated with the Ranking of Boxers. *Perceptual and Motor Skills, 119*(1), 50-58. doi:10.2466/30.29.pms.119c12z9

Buśko, K., & Gajewski, J. (2011). Muscle Strength and Power of Elite Female and Male Swimmers. *Baltic Journal of Health and Physical Activity, 3*(1), 13-18. doi:10.2478/V10131-011-0001-9

Callender, N. A., Hayes, T. N., & Tiller, N. B. (2021). Cardiorespiratory demands of competitive rock climbing. *Applied Physiology, Nutrition, and Metabolism, 46*(2), 161-168. doi:10.1139/apnm-2020-0566

Čepulėnas, A., Bružas, V., Mockus, P., & Subačius, V. (2011). Impact of physical training mesocycle on athletic and specific fitness of elite boxers. *Science of Martial Arts, 7*(1), 33-39.

Chaverri, D., Iglesias, X., Schuller, T., Hoffmann, U., & Rodríguez, F. A. (2016). Estimating peak oxygen uptake based on postexercise measurements in swimming. *Applied Physiology, Nutrition, and Metabolism, 41*(6), 588-596. doi:10.1139/apnm-2015-0524

Chiodo, S., Tessitore, A., Cortis, C., Lupo, C., Ammendolia, A., Iona, T., & Capranica, L. (2011). Effects of official Taekwondo competitions on all-out performances of elite athletes. *Journal of Strength and Conditioning Research, 25*(2), 334-339. doi:10.1519/JSC.0b013e3182027288

Clarke, J. S., Highton, J. M., Close, G. L., & Twist, C. (2019). Carbohydrate and Caffeine Improves High-Intensity Running of Elite Rugby League Interchange Players During Simulated Match Play. *Journal of Strength and Conditioning Research, 33*(5), 1320-1327. doi:10.1519/JSC.0000000000001742

Comfort, P., Graham-Smith, P., Matthews, M. J., & Bamber, C. (2011). Strength and power characteristics in English elite rugby league players. *Journal of Strength and Conditioning Research, 25*(5), 1374-1384. doi:10.1519/JSC.0b013e3181d687f5

Crewther, B. T., Kilduff, L. P., Cook, C. J., Cunningham, D. J., Bunce, P., Bracken, R. M., & Gaviglio, C. M. (2012). Relationships between salivary free testosterone and the expression of force and power in elite athletes. *Journal of Sports Medicine and Physical Fi ness, 52*(2), 221-227. Retrieved from https://www.ncbi.nlm.nih.gov/pubmed/22531608

Czuba, M., Waskiewicz, Z., Zajac, A., Poprzecki, S., Cholewa, J., & Roczniok, R. (2011). The effects of intermittent hypoxic training on aerobic capacity and endurance performance in cyclists. *Journal of Sports Science & Medicine, 10*(1), 175-183. Retrieved from https://www.ncbi.nlm.nih.gov/pubmed/24149312

Dalamitros, A. A., Mavridis, G., Semaltianou, E., Loupos, D., & Manou, V. (2019). Psychophysiological and performance-related responses of a potentiation activity in swimmers of different competitive levels. *Physiology and Behavior, 204*, 106-111. doi:10.1016/j.physbeh.2019.02.018

Dallas, G., Zacharogiannis, E., & Paradisis, G. (2013). Physiological profile of elite Greek gymnasts. *Journal of Physical Education and Sport, 13*(1). doi:10.7752/jpes.2013.01005

Das, A., Mandal, M., Majumdar, P., & Syamal, A. K. (2019). Morpho-physiological profile and 2K performance of Indian elite rowers. *Journal of Physical Education and Sport, 19*(3), 1630-1635. doi:10.7752/jpes.2019.03236

Dello Iacono, A., Martone, D., Milic, M., & Padulo, J. (2017). Vertical- vs. Horizontal-Oriented Drop Jump Training: Chronic Effects on Explosive Performances of Elite Handball Players. *Journal of Strength and Conditioning Research, 31*(4), 921-931. doi:10.1519/JSC.0000000000001555

Donti, O., Tsolakis, C., & Bogdanis, G. C. (2014). Effects of baseline levels of flexibility and vertical jump ability on performance following different volumes of static stretching and potentiating exercises in elite gymnasts. *Journal of Sports Science & Medicine, 13*(1), 105-113. Retrieved from https://www.ncbi.nlm.nih.gov/pubmed/24570613

Drid, P., Casals, C., Mekic, A., Radjo, I., Stojanovic, M., & Ostojic, S. M. (2015). Fitness and Anthropometric Profiles of International vs. National Judo Medalists in Half-Heavyweight Category. *Journal of Strength and Conditioning Research, 29*(8), 2115-2121. doi:10.1519/JSC.0000000000000861

Đurković, T., Marelić, N., & Rešetar, T. (2014). Differences in aerobic capacity indicators between the Croatian national team and club level volleyball players. *Kinesiology, 46*(1), 59-65.

El-Ashker, S., & Nasr, M. (2012). Effect of boxing exercises on physiological and biochemical responses of Egyptian elite boxers. *Journal of Physical Education and Sport, 12*(1), 111-116. doi:10.7752/jpes.2012.01018

Erdağı, K. (2020). The study of the correlations between handgrip strength and some anthropometric characteristics of upper extremity of elite and sub-elite Olympic style weightlifting athletes. *Physical education of students, 24*(1), 19-30. doi:10.15561/20755279.2020.0103

Fernandez-Fernandez, J., Sanz-Rivas, D., Sarabia, J. M., & Moya, M. (2015). Preseason Training: The Effects of a 17-Day High-Intensity Shock Microcycle in Elite Tennis Players. *Journal of Sports Science & Medicine, 14*(4), 783-791. Retrieved from https://www.ncbi.nlm.nih.gov/pubmed/26664275

Ferragut, C., Vila, H., Abraldes, J. A., Argudo, F., Rodriguez, N., & Alcaraz, P. E. (2011). Relationship among maximal grip, throwing velocity and anthropometric parameters in elite water polo players. *Journal of Sports Medicine and Physical Fi ness, 51*(1), 26-32. Retrieved from https://www.ncbi.nlm.nih.gov/pubmed/21297560

Ferragut Fiol, C., Abraldes Valeiras, J. A., Manchado López, M. d. C., & Vila Suárez, H. (2015). Water polo throwing speed and body composition: an analysis by playing positions and opposition level. *Journal of Human Sport and Exercise, 10*(1). doi:10.14198/jhse.2015.101.07

Fessi, M. S., Zarrouk, N., Filetti, C., Rebai, H., Elloumi, M., & Moalla, W. (2016). Physical and anthropometric changes during pre- and in-season in professional soccer players. *Journal of Sports Medicine and Physical Fi ness, 56*(10), 1163-1170. Retrieved from https://www.ncbi.nlm.nih.gov/pubmed/26364664

Furness, J. W., Hing, W. A., Sheppard, J. M., Newcomer, S. C., Schram, B. L., & Climstein, M. (2018). Physiological Profile of Male Competitive and Recreational Surfers. *Journal of Strength and Conditioning Research, 32*(2), 372-378. doi:10.1519/jsc.0000000000001623

Galy, O., Ben Zoubir, S., Hambli, M., Chaouachi, A., Hue, O., & Chamari, K. (2014). Relationships between heart rate and physiological parameters of performance in top-level water polo players. *Biology of Sport, 31*(1), 33-38. doi:10.5604/20831862.1083277

García-Pallarés, J., García-Fernández, M., Sánchez-Medina, L., & Izquierdo, M. (2010). Performance changes in world-class kayakers following two different training periodization models. *European Journal of Applied Physiology, 110*(1), 99-107. doi:10.1007/s00421-010-1484-9

García-Pallarés, J., López-Gullón, J. M., Muriel, X., Díaz, A., & Izquierdo, M. (2011). Physical fitness factors to predict male Olympic wrestling performance. *European Journal of Applied Physiology, 111*(8), 1747-1758. doi:10.1007/s00421-010-1809-8

Garrido, N. D., Silva, A. J., Fernandes, R. J., Barbosa, T. M., Costa, A. M., Marinho, D., & Marques, M. C. (2012). High Level Swimming Performance and its Relation to Non-Specific Parameters: A Cross-Sectional Study on Maximum Handgrip Isometric Strength. *Perceptual and Motor Skills, 114*(3), 936-948. doi:10.2466/05.10.25.pms.114.3.936-948

Ghloum, K., & Hajji, S. (2011). Comparison of diet consumption, body composition and lipoprotein lipid values of Kuwaiti fencing players with international norms. *Journal of the International Society of Sports Nutrition, 8*(1), 13. doi:10.1186/1550-2783-8-13

Ghorbanzadeh, B., Müniroglu, S., Akalan, C., Khodadadi, M. R., Kirazci, S., & Sahin, M. (2011). Determination of Taekwondo National Team Selection Criterions by Measuring Physical and Physiological Parameters. *Annals of Biological Research, 2*(6), 184-197.

Giatsis, G., Panoutsakopoulos, V., & Kollias, I. A. (2018). Biomechanical differences of arm swing countermovement jumps on sand and rigid surface performed by elite beach volleyball players. *Journal of Sports Sciences, 36*(9), 997-1008. doi:10.1080/02640414.2017.1348614

González-Parra, G., Mora, R., & Hoeger, B. (2013). Maximal oxygen consumption in national elite triathletes that train in high altitude. *Journal of Human Sport and Exercise, 8*(2), 342-349.

González-Ravé, J. M., Juárez, D., Rubio-Arias, J. A., Clemente-Suarez, V. J., Martinez-Valencia, M. A., & Abian-Vicen, J. (2014). Isokinetic Leg Strength and Power in Elite Handball Players. *Journal of Human Kinetics, 41*(1), 227-233. doi:10.2478/hukin-2014-0050

Halperin, I., Hughes, S., & Chapman, D. W. (2016). Physiological profile of a professional boxer preparing for Title Bout: A case study. *Journal of Sports Sciences, 34*(20), 1949-1956. doi:10.1080/02640414.2016.1143110

Hamano, S., Ochi, E., Tsuchiya, Y., Muramatsu, E., Suzukawa, K., & Igawa, S. (2015). Relationship between performance test and body composition/physical strength characteristic in sprint canoe and kayak paddlers. *Open Access Journal of Sports Medicine*, 191. doi:10.2147/oajsm.s82295

Helgerud, J., Rodas, G., Kemi, O. J., & Hoff, J. (2011). Strength and endurance in elite football players. *International Journal of Sports Medicine, 32*(9), 677-682. doi:10.1055/s-0031-1275742

Hermassi, S., Gabbett, T. J., Ingebrigtsen, J., Van Den Tillaar, R., Chelly, M. S., & Chamari, K. (2014). Effects of a Short-Term In-Season Plyometric Training Program on Repeated-Sprint Ability, Leg Power and Jump Performance of Elite Handball Players. *International Journal of Sports Science & Coaching, 9*(5), 1205-1216. doi:10.1260/1747-9541.9.5.1205

Hinrichs, T., Franke, J., Voss, S., Bloch, W., Schanzer, W., & Platen, P. (2010). Total hemoglobin mass, iron status, and endurance capacity in elite field hockey players. *Journal of Strength and Conditioning Research, 24*(3), 629-638. doi:10.1519/JSC.0b013e3181a5bc59

Hoffmann, M., Moeller, T., Seidel, I., & Stein, T. (2017). Predicting Elite Triathlon Performance: A Comparison of Multiple Regressions and Artificial Neural Networks. *International Journal of Computer Science in Sport, 16*(2), 101-116. doi:10.1515/ijcss-2017-0009

Horta, T. A. G., Bara Filho, M. G., Coimbra, D. R., Miranda, R., & Werneck, F. Z. (2019). Training Load, Physical Performance, Biochemical Markers, and Psychological Stress During a Short Preparatory Period in Brazilian Elite Male Volleyball Players. *Journal of Strength and Conditioning Research, 33*(12), 3392-3399. doi:10.1519/JSC.0000000000002404

Hough, J., Robertson, C., & Gleeson, M. (2015). Blunting of exercise-induced salivary testosterone in elite-level triathletes with a 10-day training camp. *International Journal of Sports Physiology and Performance, 10*(7), 935-938. doi:10.1123/ijspp.2014-0360

Ireton, M. R. E., Till, K., Weaving, D., & Jones, B. (2019). Differences in the Movement Skills and Physical Qualities of Elite Senior and Academy Rugby League Players. *Journal of Strength and Conditioning Research, 33*(5), 1328-1338. doi:10.1519/JSC.0000000000002016

Khanbabazadeh, M., Serajian, A., & Rashidlamir, A. (2016). Digit Ratio, Testosterone/Cortisol Levels, and Hand Grip Strength Among Elite Iranian Wrestlers. *International Journal of Wrestling Science, 6*(1), 53-57. doi:10.1080/21615667.2016.1197707

Khayyat, H. N., Sağır, S. G., Hataş, Ö., Smolarczyk, M., & Akalan, C. (2020). Physical, physiological and psychological profiles of elite Turkish taekwondo athletes. *Biomedical Human Kinetics, 12*(1), 187-196. doi:10.2478/bhk-2020-0024

Kitamura, K., Pereira, L. A., Kobal, R., Abad, C. C. C., Finotti, R., Nakamura, F. Y., & Loturco, I. (2017). Loaded and unloaded jump performance of top-level volleyball players from different age categories. *Biology of Sport, 3*, 273-278. doi:10.5114/biolsport.2017.67123

Klusiewicz, A., Starczewski, M., Ładyga, M., Długołęcka, B., Braksator, W., Mamcarz, A., & Sitkowski, D. (2014). Reference Values of Maximal Oxygen Uptake for Polish Rowers. *Journal of Human Kinetics, 44*(1), 121-127. doi:10.2478/hukin-2014-0117

Knab, A. M., Nieman, D. C., Gillitt, N. D., Shanely, R. A., Cialdella-Kam, L., Henson, D. A., & Sha, W. (2013). Effects of a Flavonoid-Rich Juice on Inflammation, Oxidative Stress, and Immunity in Elite Swimmers: A Metabolomics-Based Approach. *International Journal of Sport Nutrition and Exercise Metabolism, 23*(2), 150-160. doi:10.1123/ijsnem.23.2.150

Kobal, R., Nakamura, F. Y., Kitamura, K., Cal Abad, C. C., Pereira, L. A., & Loturco, I. (2017). Vertical and depth jumping performance in elite athletes from different sports specialties. *Science & Sports, 32*(5), e191-e196. doi:10.1016/j.scispo.2017.01.007

Koropanovski, N., Berjan, B., Bozic, P., Pazin, N., Sanader, A., Jovanovic, S., & Jaric, S. (2011). Anthropometric and Physical Performance Profiles of Elite Karate Kumite and Kata Competitors. *Journal of Human Kinetics, 30*(1), 107-114. doi:10.2478/v10078-011-0078-x

Kozłowska, L., Mizera, O., Gromadzińska, J., Janasik, B., Mikołajewska, K., Mróz, A., & Wąsowicz, W. (2020). Changes in Oxidative Stress, Inflammation, and Muscle Damage Markers Following Diet and Beetroot Juice Supplementation in Elite Fencers. *Antioxidants, 9*(7), 571. doi:10.3390/antiox9070571

Krommes, K., Petersen, J., Nielsen, M. B., Aagaard, P., Hölmich, P., & Thorborg, K. (2017). Sprint and jump performance in elite male soccer players following a 10-week Nordic Hamstring exercise Protocol: a randomised pilot study. *BMC Research Notes, 10*(1). doi:10.1186/s13104-017-2986-x

Krzykala, M., Leszczynski, P., Grzeskowiak, M., Podgorski, T., Wozniewicz-Dobrzynska, M., Konarska, A., . . . Konarski, J. M. (2018). Does field hockey increase morphofunctional asymmetry? A pilot study. *Homo, 69*(1-2), 43-49. doi:10.1016/j.jchb.2018.03.003

La Monica, M. B., Fukuda, D. H., Miramonti, A. A., Beyer, K. S., Hoffman, M. W., Boone, C. H., . . . Hoffman, J. R. (2016). Physical Differences Between Forwards and Backs in American Collegiate Rugby Players. *Journal of Strength and Conditioning Research, 30*(9), 2382-2391. doi:10.1519/JSC.0000000000001388

Laudner, K., Vazquez, J., Selkow, N., & Meister, K. (2017). Strong Correlation of Upper-Extremity Blood-Flow Volume With Grip Strength While in a Provocative Shoulder Position in Baseball Pitchers. *J Sport Rehabil, 26*(4), 234-237. doi:10.1123/jsr.2015-0179

Legaz-Arrese, A., Munguía-Izquierdo, D., Carranza-García, L., Reverter-Masía, J., Torres-Dávila, C., & Medina-Rodríguez, R. (2011). The validity of incremental exercise testing in discriminating of physiological profiles in elite runners. *Acta Physiologica Hungarica, 98*(2), 147-156. doi:10.1556/aphysiol.98.2011.2.6

Lijewski, M., Burdukiewicz, A., Pietraszewska, J., Stachoń, A., Andrzejewska, J., & Chromik, K. (2019). Anthropometric and strength profiles of professional handball players in relation to their playing position – multivariate analysis. *Acta of Bioengineering and Biomechanics, 21*(4). doi:10.37190/abb-01471-2019-02

Lim, C.-H., Yoon, J.-R., Jeong, C.-S., & Kim, Y.-S. (2018). An Analysis of the Performance Determinants of Modern Pentathlon Athletes in Laser-run, A Newly-Combined Event in Modern Pentathlon. *Exercise Science, 27*(1), 62-70. doi:10.15857/ksep.2018.27.1.62

Limonta, E., Brighenti, A., Rampichini, S., Cè, E., Schena, F., & Esposito, F. (2018). Cardiovascular and metabolic responses during indoor climbing and laboratory cycling exercise in advanced and élite climbers. *European Journal of Applied Physiology, 118*(2), 371-379. doi:10.1007/s00421-017-3779-6

Limonta, E., Ce, E., Gobbo, M., Veicsteinas, A., Orizio, C., & Esposito, F. (2016). Motor unit activation strategy during a sustained isometric contraction of finger flexor muscles in elite climbers. *Journal of Sports Sciences, 34*(2), 133-142. doi:10.1080/02640414.2015.1035738

Lindenthaler, J. R., Rice, A. J., Versey, N. G., McKune, A. J., & Welvaert, M. (2018). Differences in Physiological Responses During Rowing and Cycle Ergometry in Elite Male Rowers. *Frontiers in Physiology, 9*. doi:10.3389/fphys.2018.01010

Loturco, I., Artioli, G. G., Kobal, R., Gil, S., & Franchini, E. (2014). Predicting punching acceleration from selected strength and power variables in elite karate athletes: a multiple regression analysis. *Journal of Strength and Conditioning Research, 28*(7), 1826-1832. doi:10.1519/JSC.0000000000000329

Loturco, I., D'Angelo, R. A., Fernandes, V., Gil, S., Kobal, R., Cal Abad, C. C., . . . Nakamura, F. Y. (2015). Relationship between sprint ability and loaded/unloaded jump tests in elite sprinters. *Journal of Strength and Conditioning Research, 29*(3), 758-764. doi:10.1519/JSC.0000000000000660

Loturco, I., Nakamura, F. Y., Artioli, G. G., Kobal, R., Kitamura, K., Cal Abad, C. C., . . . Franchini, E. (2016). Strength and Power Qualities Are Highly Associated With Punching Impact in Elite Amateur Boxers. *Journal of Strength and Conditioning Research, 30*(1), 109-116. doi:10.1519/JSC.0000000000001075

Loturco, I., Pereira, L., Kobal, R., Cal Abad, C., Fernandes, V., Ramirez-Campillo, R., & Suchomel, T. (2018). Portable Force Plates: A Viable and Practical Alternative to Rapidly and Accurately Monitor Elite Sprint Performance. *Sports, 6*(3), 61. doi:10.3390/sports6030061

Louis, J., Billaut, F., Bernad, T., Vettoretti, F., Hausswirth, C., & Brisswalter, J. (2013). Physiological demands of a simulated BMX competition. *International Journal of Sports Medicine, 34*(6), 491-496. doi:10.1055/s-0032-1327657

Macdermid, P. W., & Stannard, S. (2012). Mechanical work and physiological responses to simulated cross country mountain bike racing. *Journal of Sports Sciences, 30*(14), 1491-1501. doi:10.1080/02640414.2012.711487

Macdonald, J. H., & Callender, N. (2011). Athletic Profile of Highly Accomplished Boulderers. *Wilderness & Environmental Medicine, 22*(2), 140-143. doi:10.1016/j.wem.2010.11.012

Manna, I., Khanna, G. L., & Dhara, P. C. (2011). Effect of Training on Anthropometric, Physiological, and Health-Related Variables of Indian Senior Elite Volleyball Players. *Asian Journal of Exercise & Sports Science, 8*(1), 64-82.

McKay, A. K. A., Heikura, I. A., Burke, L. M., Peeling, P., Pyne, D. B., van Swelm, R. P. L., . . . Cox, G. R. (2019). Influence of Periodizing Dietary Carbohydrate on Iron Regulation and Immune Function in Elite Triathletes. *International Journal of Sport Nutrition and Exercise Metabolism*, 1-8. doi:10.1123/ijsnem.2019-0131

Meckel, Y., Doron, O., Eliakim, E., & Eliakim, A. (2018). Seasonal Variations in Physical Fitness and Performance Indices of Elite Soccer Players. *Sports, 6*(1), 14. doi:10.3390/sports6010014

Mejias, J. E., Bragada, J. A., Costa, M. J., Reis, V. M., Garrido, N. D., & Barbosa, T. M. (2014). “Young” masters vs. elite swimmers: Comparison of performance,

energetics, kinematics and efficiency. *International SportMed Journal, 15*(2), 165-177. doi:10.10520/EJC154975

Menaspa, P., Martin, D. T., Victor, J., & Abbiss, C. R. (2015). Maximal Sprint Power in Road Cyclists After Variable and Nonvariable High-Intensity Exercise. *Journal of Strength and Conditioning Research, 29*(11), 3156-3161. doi:10.1519/JSC.0000000000000972

Michalsik, L. B., Madsen, K., & Aagaard, P. (2015). Physiological capacity and physical testing in male elite team handball. *Journal of Sports Medicine and Physical Fi ness, 55*(5), 415-429. Retrieved from https://www.ncbi.nlm.nih.gov/pubmed/24402441

Mikulic, P. (2011). Maturation to elite status: a six-year physiological case study of a world champion rowing crew. *European Journal of Applied Physiology, 111*(9), 2363-2368. doi:10.1007/s00421-011-1870-y

Mon, D., Zakynthinaki, M. S., Cordente, C. A., Antón, A. J. M., Rodríguez, B. R., & Jiménez, D. L. (2015). Finger Flexor Force Influences Performance in Senior Male Air Pistol Olympic Shooting. *PloS One, 10*(6), e0129862. doi:10.1371/journal.pone.0129862

Morán-Navarro, R., Valverde-Conesa, A., López-Gullón, J. M., De la Cruz-Sánchez, E., & Pallarés, J. G. (2015). Can balance skills predict olympic wrestling performance? *Journal of Sport and Health Research, 7*(1), 19-30.

Moro, T., Tinsley, G., Longo, G., Grigoletto, D., Bianco, A., Ferraris, C., . . . Paoli, A. (2020). Time-restricted eating effects on performance, immune function, and body composition in elite cyclists: a randomized controlled trial. *Journal of the International Society of Sports Nutrition, 17*(1). doi:10.1186/s12970-020-00396-z

Najmi, N., Abdullah, M. R., Juahir, H., Maliki, A. B. H. M., Musa, R. M., Mat-Rasid, S. M., . . . Alias, N. (2018). COMPARISON OF BODY FAT PERCENTAGE AND PHYSICAL PERFORMANCE OF MALE NATIONAL SENIOR AND JUNIOR KARATE ATHLETES. *Journal of Fundamental and Applied Sciences, 10*(1S), 485-511. doi:10.4314/jfas.v10i1s

Nevill, A. M., Allen, S. V., & Ingham, S. A. (2011). Modelling the determinants of 2000 m rowing ergometer performance: a proportional, curvilinear allometric approach. *Scand J Med Sci Sports, 21*(1), 73-78. doi:10.1111/j.1600-0838.2009.01025.x

Nikooie, R., Cheraghi, M., & Mohamadipour, F. (2017). Physiological determinants of wrestling success in elite Iranian senior and junior Greco-Roman wrestlers. *Journal of Sports Medicine and Physical Fi ness, 57*(3), 219-226. doi:10.23736/S0022-4707.16.06017-5

Nilsson, J., & Cardinale, D. (2015). Aerobic and anaerobic test performance among elite male football players in different team positions. *LASE Journal of Sport Science, 6*(2), 73-92.

Paoli, A., Grimaldi, K., D’Agostino, D., Cenci, L., Moro, T., Bianco, A., & Palma, A. (2012). Ketogenic diet does not affect strength performance in elite artistic gymnasts. *Journal of the International Society of Sports Nutrition, 9*(1), 34. doi:10.1186/1550-2783-9-34

Parker, J., Lagerhem, C., Hellström, J., & Olsson, M. C. (2017). Effects of nine weeks isokinetic training on power, golf kinematics, and driver performance in pre-elite golfers. *BMC Sports Science, Medicine and Rehabilitation, 9*(1). doi:10.1186/s13102-017-0086-9

Peeling, P., Cox, G. R., Bullock, N., & Burke, L. M. (2015). Beetroot Juice Improves On-Water 500 M Time-Trial Performance, and Laboratory-Based Paddling Economy in National and International-Level Kayak Athletes. *International Journal of Sport Nutrition and Exercise Metabolism, 25*(3), 278-284. doi:10.1123/ijsnem.2014-0110

Peña, J., Moreno-Doutres, D., Coma, J., Cook, M., & Buscà, B. (2018). Anthropometric and fitness profile of high-level basketball, handball and volleyball players. *Revista Andaluza de Medicina del Deporte, 11*(1), 30-35. doi:10.1016/j.ramd.2016.03.002

Philippe, K., Paillard, T., Dubois, R., Maurelli, O., & Prioux, J. (2020). Key performance indicators in Tour de France sailing. *Journal of Sports Sciences*, 1-11. doi:10.1080/02640414.2020.1851925

Philpott, L. K., Forrester, S. E., Van Lopik, K. A., Hayward, S., Conway, P. P., & West, A. A. (2020). Countermovement jump performance in elite male and female sprinters and high jumpers. *Proceedings of the Institution of Mechanical Engineers, Part P: Journal of Sports Engineering and Technology*, 175433712097143. doi:10.1177/1754337120971436

Prommer, N., Thoma, S., Quecke, L., Gutekunst, T., Volzke, C., Wachsmuth, N., . . . Schmidt, W. (2010). Total hemoglobin mass and blood volume of elite Kenyan runners. *Medicine and Science in Sports and Exercise, 42*(4), 791-797. doi:10.1249/MSS.0b013e3181badd67

Ramirez-Campillo, R., Alvarez, C., Henriquez-Olguin, C., Baez, E. B., Martinez, C., Andrade, D. C., & Izquierdo, M. (2014). Effects of plyometric training on endurance and explosive strength performance in competitive middle- and long-distance runners. *Journal of Strength and Conditioning Research, 28*(1), 97-104. doi:10.1519/JSC.0b013e3182a1f44c

Ramos Veliz, R., Requena, B., Suarez-Arrones, L., Newton, R. U., & Saez de Villarreal, E. (2014). Effects of 18-week in-season heavy-resistance and power training on throwing velocity, strength, jumping, and maximal sprint swim performance of elite male water polo players. *Journal of Strength and Conditioning Research, 28*(4), 1007-1014. doi:10.1519/JSC.0000000000000240

Randell, R. K., Carter, J. M., Jeukendrup, A. E., Lizarraga, M. A., Yanguas, J. I., & Rollo, I. (2019). Fat Oxidation Rates in Professional Soccer Players. *Medicine and Science in Sports and Exercise, 51*(8), 1677-1683. doi:10.1249/MSS.0000000000001973

Requena, B., Garcia, I., Suarez-Arrones, L., Saez de Villarreal, E., Naranjo Orellana, J., & Santalla, A. (2017). Off-Season Effects on Functional Performance, Body Composition, and Blood Parameters in Top-Level Professional Soccer Players. *Journal of Strength and Conditioning Research, 31*(4), 939-946. doi:10.1519/JSC.0000000000001568

Rodriguez, F. A., Iglesias, X., Feriche, B., Calderon-Soto, C., Chaverri, D., Wachsmuth, N. B., . . . Levine, B. D. (2015). Altitude Training in Elite Swimmers for Sea Level Performance (Altitude Project). *Medicine and Science in Sports and Exercise, 47*(9), 1965-1978. doi:10.1249/MSS.0000000000000626

Ronnestad, B. R., Hansen, J., & Nygaard, H. (2017). 10 weeks of heavy strength training improves performance-related measurements in elite cyclists. *Journal of Sports Sciences, 35*(14), 1435-1441. doi:10.1080/02640414.2016.1215499

Saez de Villarreal, E., Suarez-Arrones, L., Requena, B., Haff, G. G., & Ramos Veliz, R. (2015). Enhancing performance in professional water polo players: dryland training, in-water training, and combined training. *Journal of Strength and Conditioning Research, 29*(4), 1089-1097. doi:10.1519/JSC.0000000000000707

Saiful Annur, M. S., Yasin, A., Syarif, A. F., & Md Radzi, N. A. A. (2017). Comparison on Selected Fitness Indicator and Golf Performances among Elite and Non-Elite University Golfers. *Social and Management Research Journal, 14*(1), 97. doi:10.24191/smrj.v14i1.5314

Santos-Concejero, J., Billaut, F., Grobler, L., Oliván, J., Noakes, T. D., & Tucker, R. (2015). Maintained cerebral oxygenation during maximal self-paced exercise in elite Kenyan runners. *Journal of Applied Physiology, 118*(2), 156-162. doi:10.1152/japplphysiol.00909.2014

Santos-Concejero, J., Oliván, J., Maté-Muñoz, J. L., Muniesa, C., Montil, M., Tucker, R., & Lucia, A. (2015). Gait-Cycle Characteristics and Running Economy in Elite Eritrean and European Runners. *International Journal of Sports Physiology and Performance, 10*(3), 381-387. doi:10.1123/ijspp.2014-0179

Secomb, J. L., Nimphius, S., Farley, O. R., Lundgren, L., Tran, T. T., & Sheppard, J. M. (2016). Lower-Body Muscle Structure and Jump Performance of Stronger and Weaker Surfing Athletes. *International Journal of Sports Physiology and Performance, 11*(5), 652-657. doi:10.1123/ijspp.2015-0481

Shalfawi, S. A., Sabbah, A., Kailani, G., Tønnessen, E., & Enoksen, E. (2011). The Relationship Between Running Speed and Measures of Vertical Jump in Professional Basketball Players: A Field-Test Approach. *Journal of Strength and Conditioning Research, 25*(11), 3088-3092. doi:10.1519/jsc.0b013e318212db0e

Silva, A. M., Fields, D. A., Heymsfield, S. B., & Sardinha, L. B. (2011). Relationship between changes in total-body water and fluid distribution with maximal forearm strength in elite judo athletes. *Journal of Strength and Conditioning Research, 25*(9), 2488-2495. doi:10.1519/JSC.0b013e3181fb3dfb

Sterkowicz-Przybycień, K., Sterkowicz, S., Biskup, L., Żarów, R., Kryst, Ł., & Ozimek, M. (2019). Somatotype, body composition, and physical fitness in artistic gymnasts depending on age and preferred event. *PloS One, 14*(2), e0211533. doi:10.1371/journal.pone.0211533

Støa, E. M., Helgerud, J., Rønnestad, B. R., Hansen, J., Ellefsen, S., & Støren, Ø. (2020). Factors Influencing Running Velocity at Lactate Threshold in Male and Female Runners at Different Levels of Performance. *Frontiers in Physiology, 11*. doi:10.3389/fphys.2020.585267

Stojanovic, M. D., Ostojic, S. M., Calleja-Gonzalez, J., Milosevic, Z., & Mikic, M. (2012). Correlation between explosive strength, aerobic power and repeated sprint ability in elite basketball players. *Journal of Sports Medicine and Physical Fi ness, 52*(4), 375-381. Retrieved from https://www.ncbi.nlm.nih.gov/pubmed/22828459

Tam, E., Rossi, H., Moia, C., Berardelli, C., Rosa, G., Capelli, C., & Ferretti, G. (2012). Energetics of running in top-level marathon runners from Kenya. *European Journal of Applied Physiology, 112*(11), 3797-3806. doi:10.1007/s00421-012-2357-1

Thiel, C., Vogt, L., Bürklein, M., Rosenhagen, A., Hübscher, M., & Banzer, W. (2011). Functional Overreaching During Preparation Training of Elite Tennis Professionals. *Journal of Human Kinetics, 28*(1). doi:10.2478/v10078-011-0025-x

Tokatlidou, C., Xirouchaki, C. E., Armenis, E., & Apostolidis, N. (2020). Hematologic, biochemical, and physiologic characteristics of elite and professional basketball players. *Journal of Physical Education and Sport, 20*(6), 3384-3390.

Tsolakis, C., & Bogdanis, G. C. (2012). Acute effects of two different warm-up protocols on flexibility and lower limb explosive performance in male and female high level athletes. *Journal of Sports Science & Medicine, 11*(4), 669-675. Retrieved from https://www.ncbi.nlm.nih.gov/pubmed/24150077

Wagner, H., Fuchs, P. X., & von Duvillard, S. P. (2018). Specific physiological and biomechanical performance in elite, sub-elite and in non-elite male team handball players. *Journal of Sports Medicine and Physical Fi ness, 58*(1-2), 73-81. doi:10.23736/S0022-4707.16.06758-X

West, D. J., Owen, N. J., Cunningham, D. J., Cook, C. J., & Kilduff, L. P. (2011). Strength and power predictors of swimming starts in international sprint swimmers. *Journal of Strength and Conditioning Research, 25*(4), 950-955. doi:10.1519/JSC.0b013e3181c8656f

Yang, S. W. (2014). Assessment of professional baseball players aerobic exercise performance depending on their positions. *Journal of Strength and Conditioning Research, 28*(11), 3289-3292. doi:10.1519/JSC.0000000000000527

Zabala, M., Peinado, A. B., Calderón, F. J., Sampedro, J., Castillo, M. J., & Benito, P. J. (2011). Bicarbonate ingestion has no ergogenic effect on consecutive all out sprint tests in BMX elite cyclists. *European Journal of Applied Physiology, 111*(12), 3127-3134. doi:10.1007/s00421-011-1938-8

Zagatto, A. M., Papoti, M., Dos Reis, I. G. M., Beck, W. R., & Gobatto, C. A. (2014). Analysis of cardiopulmonary and metabolic variables measured during laboratory and sport-specific incremental tests for table tennis performance prediction. *29*(2), 62-70. doi:10.1016/j.scispo.2013.08.001

Zaggelidis, G. (2016). Maximal Isometric Handgrip Strength (HGS) in Greek Elite Male Judo and Karate Athletes. *Sport Science Review, 25*(5-6), 320-333. doi:10.1515/ssr-2016-0017

Zinner, C., Jakobs, K., Sperlich, B., Wahl, P., & Mester, J. (2010). *100 m personal best sprint time does not correlate with VO2max in elite sprinters*. Paper presented at the 15th Annual Congress of the European College of Sport Science, Antalya, Turkey.

Zouhal, H., Le Douairon Lahaye, S., Ben Abderrahaman, A., Minter, G., Herbez, R., & Castagna, C. (2012). Energy system contribution to Olympic distances in flat water kayaking (500 and 1,000 m) in highly trained subjects. *Journal of Strength and Conditioning Research, 26*(3), 825-831. doi:10.1519/JSC.0b013e31822766f7
